# Vitamin B12 promotes cefiderocol resistance and small-colony variants in carbapenem-resistant *Acinetobacter baumannii*

**DOI:** 10.1128/mbio.03760-25

**Published:** 2026-01-16

**Authors:** Vyanka Mezcord, Irene Luu, Usman Akhar, German M. Traglia, Cecilia Rodríguez, Samyar Moheb, Shayra D. Sanchez, Maria T. Soto, Maria J. Cima Clave, Rodrigo Sieira, Marisel R. Tuttobene, Alejandra Corso, Marcelo E. Tolmasky, Robert A. Bonomo, Luis A. Actis, Gauri Rao, Fernando Pasteran, Maria S. Ramirez

**Affiliations:** 1 Center for Applied Biotechnology Studies, Department of Biological Science, College of Natural Sciences and Mathematics, California State University Fullerton 118567, Fullerton, California, USA; 2 Unidad de Genómica y Bioinformática, Departamento de Ciencias Biológicas, CENUR Litoral Norte, Universidad de la República 113082 , Montevideo, Uruguay; 3 Centro de Referencia para Lactobacilos (CERELA), CONICET 647819 , Tucumán, Argentina; 4 Laboratorio Nacional/Regional de Referencia en Antimicrobianos, Instituto Nacional de Enfermedades Infecciosas, ANLIS Dr. Carlos G. Malbrán 62986 , Buenos Aires, Argentina; 5 Fundación Instituto Leloir-IIBBA CONICET 62898, Buenos Aires, Argentina; 6 Instituto de Biología Molecular y Celular de Rosario (IBR, CONICET-UNR) 63031, Rosario, Argentina; 7 Research Service and GRECC, Louis Stokes Cleveland Department of Veterans Affairs Medical Center 20083, Cleveland, Ohio, USA; 8 Departments of Medicine, Case Western Reserve University School of Medicine 12304, Cleveland, Ohio, USA; 9 Department of Pharmacology, Case Western Reserve University School of Medicine 12304, Cleveland, Ohio, USA; 10 Department of Molecular Biology and Microbiology, Case Western Reserve University School of Medicine 12304, Cleveland, Ohio, USA; 11 Department of Biochemistry, Case Western Reserve University School of Medicine 12304, Cleveland, Ohio, USA; 12 Department of Proteomics and Bioinformatics, Case Western Reserve University School of Medicine 12304, Cleveland, Ohio, USA; 13 CWRU-Cleveland VAMC Center for Antimicrobial Resistance and Epidemiology (Case VA CARES) 14666, Cleveland, Ohio, USA; 14 Department of Microbiology, Miami University 6403, Oxford, Ohio, USA; 15 University of Southern California 5116, Los Angeles, California, USA; McMaster University, Hamilton, Ontario, Canada

**Keywords:** *Acinetobacter baumannii*, cefiderocol, vitamin B12, TonB-dependent receptors, small-colony variants, antibiotic resistance

## Abstract

Carbapenem-resistant *Acinetobacter baumannii* (CRAB) is a global health threat with few effective treatment options remaining. Cefiderocol, a last-resort siderophore-cephalosporin antibiotic, exploits bacterial iron transport systems via TonB-dependent receptors (TBDRs) to gain cellular entry. However, treatment failures and the emergence of resistance highlight concerns with *in vivo* efficacy. In this study, we report an unanticipated cefiderocol resistance mechanism where vitamin B12, a micronutrient supplement, modulates cefiderocol susceptibility. Our work revealed that vitamin B12 (methylcobalamin) affects and interacts with TBDRs and other metabolic and adaptation processes that contribute to increased cefiderocol MIC levels and the emergence of persistence phenotypes. We demonstrate that vitamin B12 supplementation elicits strain-specific transcriptomic responses in the AB5075 and AMA17 CRAB strains, characterized by the downregulation of genes encoding siderophore-mediated iron acquisition functions, stress responses, metabolic reprogramming, and biofilm biogenesis. Structural modeling and molecular docking reveal overlapping binding sites for vitamin B12 and cefiderocol within TBDRs such as CirA and PirA, suggesting competitive inhibition. Additionally, vitamin B12 exposure increases cefiderocol MICs across a panel of *A. baumannii* clinical and reference strains, enhances survival in time-kill assays, and promotes the emergence of small-colony variants displaying persistent phenotypes. Notably, this effect is stable, dose dependent, and further enhanced in the presence of host-derived fluids. These findings describe a previously unrecognized host–pathogen–drug interaction with potential clinical implications, suggesting that vitamin B12 exposure could contribute to cefiderocol treatment failure. Our results underscore the urgent need to consider the potential contribution of vitamin supplements to antimicrobial therapy and management strategies for CRAB infections.

**IMPORTANCE:**

Cefiderocol, a last-line antibiotic for treating carbapenem-resistant *Acinetobacter baumannii* (CRAB) infections, uses iron-uptake receptors to enter bacterial cells. Our work demonstrates that vitamin B12, a common supplement in outpatients and hospitalized adults, can antagonize cefiderocol by affecting TonB-dependent receptor expression and competing at shared entry sites. As a result, cefiderocol MICs are raised, thus promoting persistent small-colony variants. This dose-dependent, strain-specific effect is amplified by host fluids, revealing a clinically plausible pathway leading to treatment failure that current susceptibility testing assays do not consider. Recognizing vitamin B12 exposure and incorporating physiological B12/iron conditions into antimicrobial susceptibility testing and models could improve decision-making for treatment regimens. More broadly, our findings highlight nutrient–antibiotic interactions as overlooked drivers of CRAB’s resistance and persistence.

## INTRODUCTION

Carbapenem-resistant *Acinetobacter baumannii* (CRAB) is a recognized nosocomial pathogen associated with ventilator-associated pneumonia, bloodstream infections, and complicated urinary tract infections, particularly in critically ill patients (1–3). Global surveillance data from the CDC and WHO indicate persistently high carbapenem resistance rates, often exceeding 50%, in regions such as Latin America, Europe, and Asia-Pacific (4–7). Therapeutic options are increasingly limited due to multidrug resistance, with *A. baumannii* commonly displaying resistance to carbapenems, β-lactams, aminoglycosides, and fluoroquinolones (1, 8, 9). Carbapenemases of the oxacillinase family, typically OXA-23, have become globally distributed among CRAB isolates (10, 11). Recently, there has been an increase in the emergence of dual-carbapenemase producers (e.g., OXA-23 and NDM-1 co-harboring strains), which further complicates treatment and infection control efforts (12–15). *A. baumannii* is also recognized for rapidly acquiring resistance to multiple antibiotics, including last-line antimicrobial agents such as the recently approved cefiderocol and sulbactam/durlobactam (13, 16–20).

Cefiderocol, a siderophore cephalosporin, offers a novel mechanism of action by utilizing the bacterial iron-uptake machinery (21–25). The catechol moiety of cefiderocol mimics natural siderophores, allowing the drug to utilize TonB-dependent receptors (TBDRs) for active transport into the periplasmic space. This “Trojan horse” strategy improves drug penetration and provides potent *in vitro* activity against CRAB isolates and other gram-negative pathogens (17, 26, 27). However, clinical outcomes with cefiderocol have been variable. The CREDIBLE-CR trial reported higher mortality rates in patients with CRAB infections treated with cefiderocol compared to the best available therapy, raising concerns about its efficacy in specific clinical contexts (17). Growing evidence suggests that mutations in TBDRs or components of the TonB energy transduction system can interfere with cefiderocol uptake, reducing antibiotic susceptibility (28–31). Some TBDR proteins were initially characterized for their specific role in iron and vitamin B12 acquisition but are now known to transport a broader range of substrates (32, 33). Thus, conditions that alter TBDR production or saturate its biological function may ultimately influence cefiderocol activity.

Vitamin B12 (cobalamin) is essential to host and bacterial systems. It supports human red blood cell production, DNA synthesis, and methylation and is required for key metabolic pathways in bacteria (34). In the intensive care unit, vitamin B12 supplementation is common due to patient deficiency (35). However, little is known about how vitamin B12 may impact bacterial physiology or antibiotic susceptibility. Recent studies with *Vibrio campbellii*, a facultative vitamin B12 consumer, demonstrated that vitamin supplementation enhances the expression of virulence factors, including genes associated with toxin synthesis, motility, and Type VI secretion systems, underscoring vitamin B12’s potential to modulate bacterial pathogenicity beyond its canonical functions (36). Bacterial vitamin B12 uptake involves the BtuB TBDR receptor, the periplasmic binding protein BtuF, and the inner membrane BtuCD transporter (37). BtuB transport activity depends on the TonB-ExbB-ExbD complex, which transduces the energy required for the active translocation of substrates into gram-negative bacteria (37). Given that this pathway is shared with catecholate-type siderophores, it is plausible that vitamin B12 may compete with cefiderocol for uptake via TonB-coupled outer membrane receptors.

Previous work has shown that excess vitamin B12 can reshape host–microbiota interactions and favor colonization by enteric pathogens in mice (38). In humans, the recommended daily intake for healthy adults is approximately 2.4 μg/day; however, critically ill patients frequently receive repeated parenteral or enteral high-dose vitamin B12 supplementation, without any clearly defined dose-limiting toxicity. These practices make transient supraphysiological vitamin B12 levels possible, particularly in severely ill patients, the same population in which cefiderocol will be an option for difficult-to-treat bacterial infections. Within this context, it is important to consider whether vitamin B12 might modulate cefiderocol activity through related TonB-dependent uptake pathways.

While studying the impact of host-derived fluids on carbapenemase-producing *Escherichia coli*, we unexpectedly identified spontaneous cefiderocol-resistant mutants with mutations in the vitamin B12 receptor gene (*btuB*) following exposure to human pleural fluid (HPF). This unanticipated observation prompted us to determine whether the exposure to vitamin B12 would similarly impact cefiderocol susceptibility in *A. baumannii* and modulate additional adaptive traits, such as biofilm formation and the emergence of small-colony variants (SCVs), phenotypes associated with persistence and antibiotic tolerance (39). Thus, the present study aims to investigate the physiological impact of vitamin B12 exposure on *A. baumannii*, revealing a previously unappreciated vitamin B12-pathogen interaction that may influence *A. baumannii* behavior, which could lead to the development of resistance or reduced efficacy of last-line antibiotics, such as cefiderocol.

## RESULTS

### A mutation in a vitamin B12 receptor leads to cefiderocol resistance in *E. coli* after HPF exposure

**Fig 1:**
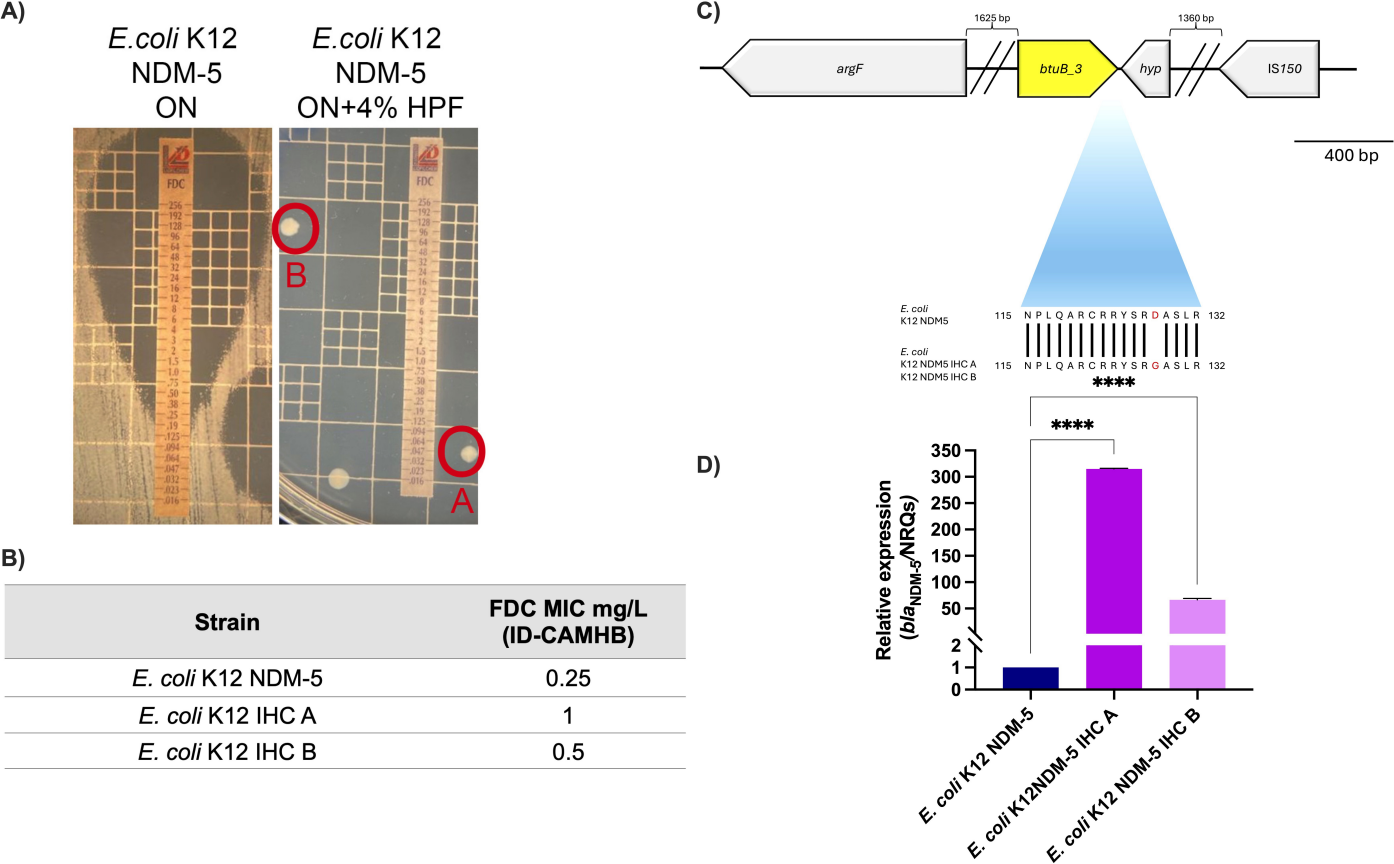
(**A**) Emergence of cefiderocol (FDC)-resistant colonies from *E. coli* K12 NDM-5 after exposure to human pleural fluid (HPF). (**B**) FDC MIC of the two resistant isolates, IHCA and IHCB, with respect to the parental strain. (**C**) Whole-genome sequencing revealed a non-synonymous D128G mutation in the *btuB_3* gene, encoding a predicted vitamin B12 TonB-dependent outer membrane receptor. (**D**) qRT-PCR analysis of *bla*_NDM-5_ transcription in the resistant mutants compared to the parental strain. The data presented are the mean ± standard deviation (SD) of normalized relative quantities (NRQs) derived from transcript levels calculated using the qBASE method. Statistical significance (*P* < 0.05) was determined by one-way ANOVA followed by Tukey’s multiple-comparison test. ****, *P* < 0.0001. ns, not significant. Technical triplicates using three independent biological samples were used to collect experimental data. Error bars represent SDs.

When investigating the effects of host-derived fluids on cefiderocol susceptibility, we exposed an *E. coli* K12 laboratory strain containing a plasmid coding for NDM-5 carbapenemase (*E. coli* K12 NDM-5) to HPF. This condition resulted in complete growth inhibition, except for the paradoxical emergence of resistant colonies. The two *E. coli* K12 NDM-5 selected emergent colonies (IHCA and IHCB) demonstrated a MIC value that was elevated by two- to fourfold dilutions compared to the parental strain (Fig. 1A and B). The whole-genome sequencing (WGS) of these resistant mutants identified 11 mutations (Table S1). While nine of these mutations mapped within intergenic or non-coding regions, two of them were located within coding regions. One of these latter mutations resulted in a non-synonymous D128G change mapped within the *btuB*_3 gene, which encodes a predicted TonB-dependent vitamin B12 outer membrane receptor (Fig. 1C and Table S1). This observation was particularly intriguing because cefiderocol also utilizes TBDRs to enter gram-negative bacterial cells, mimicking natural iron chelators like siderophores. Based on our sequence and structural analyses (see below), we propose that the D128G substitution may alter BtuB_3-mediated transport of vitamin B12 and siderophore-like molecules such as cefiderocol, thereby contributing to the altered cefiderocol susceptibility observed in these mutants.

To further study the causes of the cefiderocol resistance in the emergent resistant colonies, the expression of *bla*_NDM-5_, known to be linked with increases in cefiderocol MICs when overexpressed, was tested (40, 41). Significant upregulation of *bla*_NDM-5_ transcript levels (8.3- and 6.05-fold increases, respectively) was observed in both resistant colonies compared to the parental strain (Fig. 1D). Notably, *bla*_NDM-5_ copy number and its immediate genetic environment were unchanged in the intrahalo isolates; however, *bla*_NDM-5_ transcripts were clearly increased. This pattern suggests that subtle regulatory alterations (potentially involving intergenic regions or trans-acting regulators elsewhere in the genome) enhance NDM-5 production. The BtuB_3 mutation and high levels of *bla*_NDM-5_ transcription can further support the emergence of cefiderocol-resistant mutants when exposed to host fluid, as reported before for CRAB strains (31, 42).

These findings prompted further investigation into the potential molecular basis underlying the interaction between vitamin B12 and cefiderocol in *A. baumannii*. We aimed to assess whether vitamin B12 exposure alters the expression of genes coding for TBDRs, iron acquisition systems, or stress response pathways that could lead to an increase in antibiotic resistance and/or modulate cefiderocol uptake and activity in *A. baumannii*. For this purpose, we studied two carbapenem-resistant (CR) model strains, AB5075 and AMA17. These strains harbor different carbapenemase genes*—bla*_OXA-23_ and *bla*_NDM-1_, respectively—and belong to distinct clonal lineages isolated from unrelated geographic regions and clinical sources at different times (Table S2).

### Structural and protein-sequence homology analyses suggest overlapping substrate specificity among TBDRs for iron and vitamin B12

**Fig 2:**
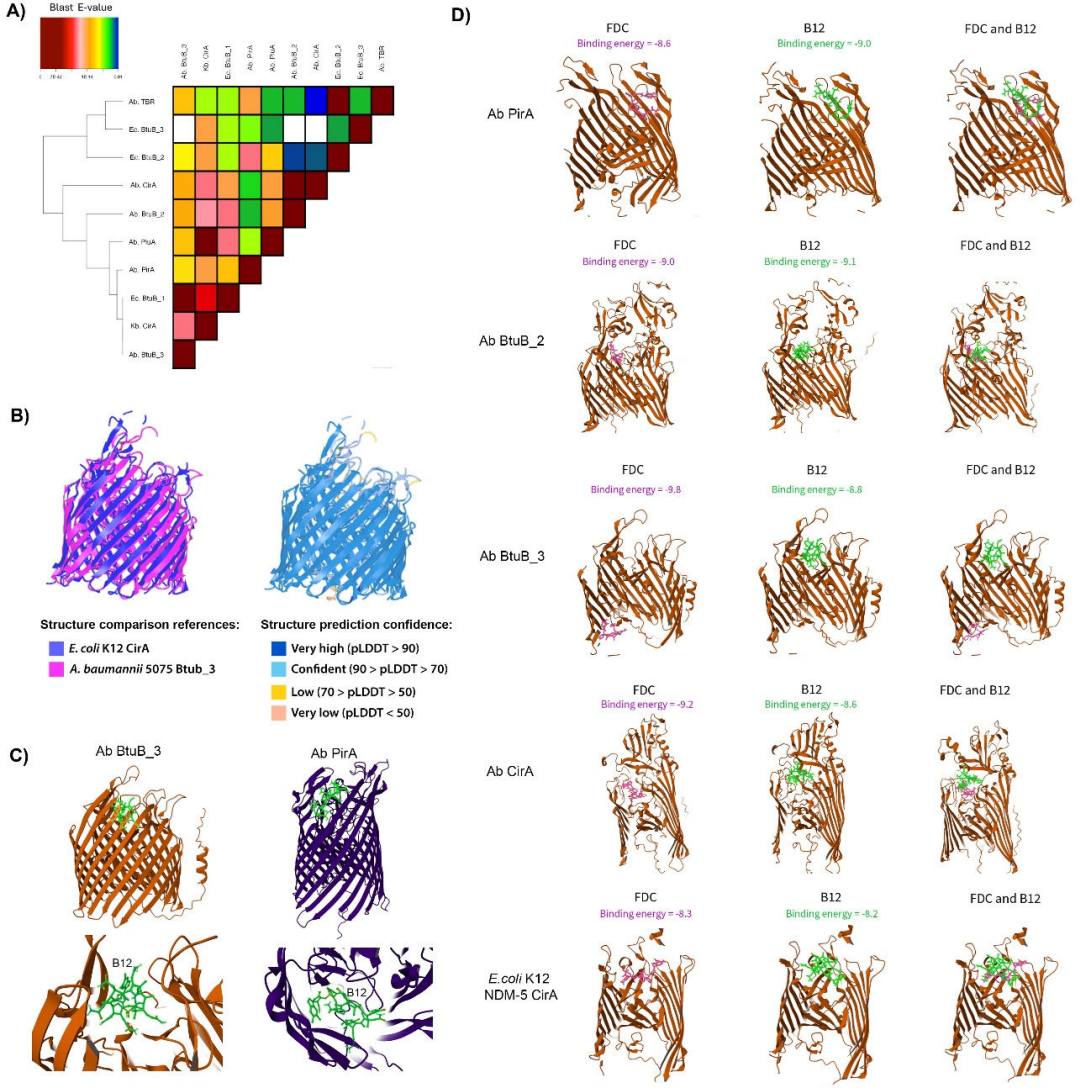
(**A**) Phylogenetic clustering of TBDRs involved in vitamin B12 and iron uptake. Heatmap illustrating protein-sequence similarity among *E. coli*, *K. pneumoniae*, and *A. baumannii* orthologs. (**B**) Structural alignment of BtuB_3 from *A. baumannii* (UniProt ID: A0A0D5YDN7) and CirA from *E. coli* K12 (UniProt ID: P17315). Structures were predicted by AlphaFold and aligned with TM-align in iCn3D. The left panel shows BtuB_3 in pink and CirA in purple, highlighting overall structural similarity. The right panel displays the same alignment colored by pLDDT confidence scores (light blue: pLDDT 70-90), indicating that alignment occurs in regions of high prediction confidence. The alignment resulted in a root-mean-square deviation of 3.145 Å and a TM score of 0.8426. (**C**) Molecular docking of vitamin B12 (neon green) into two *A. baumannii* TonB-dependent receptors using AutoDock Vina within the HotSpot Wizard platform, visualized with Mol* 3D Viewer. (Left) BtuB_3 complex with interacting residues within 3 Å identified by ChimeraX (V88, S90, T96, S98, S106, T416, D419, S423, S466, and R529); (right) PirA complex with interacting residues E100, R343, D527, N528, S528, T531, E542, Q577, P580, N662, R627, and P643. (**D**) Molecular docking of cefiderocol (FDC, purple) and vitamin B12 (green) into TBDRs from *A. baumannii* (BtuB_3, BtuB_2, PirA, and CirA) and *E. coli* (CirA). Docking was performed using HotSpot Wizard v.3.1 and visualized in Mol* 3D Viewer. The binding energies (kcal/mol) in the extracellular loop above the plug domain have been predicted and depicted in pink or green for FDC and vitamin B12, respectively.

To investigate whether cefiderocol entry and vitamin B12 uptake converge at the receptor level, we conducted a comparative sequence and structure-based analysis of *A. baumannii* and *E. coli* TBDRs*,* as well as the well-characterized iron transporter CirA from *Klebsiella pneumoniae*, with a specific focus on receptors involved in iron and B12 transport. The *E. coli* BtuB_3-predicted protein showed significant amino acid sequence homology with most B12 and iron TBDRs forming a cluster with *E. coli* BtuB_2 and other non-characterized TBDR from *A. baumannii* (Fig. 2A and Table S1). Another cluster included *A. baumannii* CirA, BtuB_2, and other iron receptor proteins, such as *A. baumannii* PirA and PiuA, which have not been fully characterized in this pathogen (Fig. 2A). Notably, in the subcluster, a higher homology was seen between *A. baumannii* BtuB_3 with *E. coli* BtuB_1 and *K. pneumoniae* CirA (Fig. 2A).

AlphaFold-predicted 3D structures, together with structural overlays, showed that the *E. coli* CirA and the *A. baumannii* AB5075 BtuB_3 proteins share highly similar β-barrel folds despite their differing primary ligands (TM score = 0.84, root-mean-square deviation [RMSD] = 3.145 Å) (Fig. 2B). Based on these observations, we determined the potential interaction of B12 with the *A. baumannii* AB5075 BtuB_3 and PirA proteins. This analysis showed that B12 could interact with both TBDRs (Fig. 2C and Table S3).

Further CirA molecular docking revealed a common binding pattern in the extracellular loop domain, where cefiderocol and vitamin B12 exhibited overlapping interaction sites. In *A. baumannii*, CirA showed a predicted binding energy that slightly favored cefiderocol over B12, with a binding energy difference of 0.6 kcal/mol (Fig. 2D). In the predicted CirA ligand-binding pocket, residues Tyr890 (backbone O), Gly842 (backbone O), and Val892 (backbone N) switched from forming hydrogen bonds with vitamin B12 to making hydrophobic contacts with cefiderocol (Fig. S1). These interaction shifts suggest a ligand-dependent rearrangement of the CirA pocket that selectively accommodates cefiderocol in *A. baumannii*. Similar binding interactions were observed for *K. pneumoniae* with an energy difference of 0.9 kcal/mol (Fig. S2). In contrast, the *E. coli* CirA showed nearly equivalent binding affinity for both ligands, with a binding energy difference of 0.1 kcal/mol (Fig. 2D). A similar shift from hydrogen bonding to hydrophobic interaction was also observed in nearby residues of *E. coli* CirA above the plug domain; Asp95 (side chain O), Lys419 (side chain N), and Glu41 (side chain O) formed hydrogen bonds in the presence of vitamin B12 while forming hydrophobic interactions when cefiderocol was bound (Fig. S1). This suggested competition for receptor binding by possibly inducing conformational changes favoring one ligand over the other. Notably, Arg116 participated in hydrogen bonding with both vitamin B12 and cefiderocol when docked above the plug domain for each ligand (Fig. S1 and S3), highlighting the importance of this residue for potential vitamin B12 or cefiderocol binding in *E. coli* CirA. Taken together, these results suggest that vitamin B12 and cefiderocol share overlapping binding sites within the CirA receptor across bacterial species. This overlap implies that, at equimolar concentrations, the similar binding affinities may limit cefiderocol’s competitive binding advantage and the ability to gain cell entry when in the presence of vitamin B12.

As observed with CirA, the *A. baumannii* PirA receptor binding sites for vitamin B12 overlapped with those of cefiderocol. Notably, PirA displayed a slight preference in binding affinity for vitamin B12 compared to cefiderocol, resulting in an energy difference of 0.4 kcal/mol (Fig. 2D), suggesting that competition may occur between vitamin B12 and cefiderocol, potentially reducing the drug’s intracellular uptake. Both vitamin B12 and cefiderocol share a common hydrogen bond to Ser530 (side chain O) (Fig. S1). Similarly, the *A. baumannii* AB5075 BtuB_2-predicted protein exhibited an overlapping binding pattern for both ligands, resulting in a binding energy difference of 0.1 kcal/mol. The residue Gln696 (side chain N) in the BtuB_2 receptor forms a hydrogen bond with both cefiderocol and vitamin B12 ligands (Fig. S1).

Comparative docking analysis of the well-characterized *E. coli* B12 uptake receptor and its *A. baumannii* AB5075 BtuB_3 ortholog revealed similar binding patterns for cefiderocol, which localize deeper within the plug domain of both receptors (Fig. 2D; Fig. S3). In contrast, vitamin B12 consistently bound to the extracellular loop domain in both *E. coli* and *A. baumannii* (Fig. 2D; Fig. S3). This vitamin B12 binding site has been previously established as the primary interaction region for TonB-dependent receptor-mediated uptake (43, 44). These findings suggest a potential for partial substrate overlap or functional redundancy between vitamin B12 and iron-siderophore TBDRs. This raises the possibility that mutations or modulation in one receptor type (e.g., *btuB_3*) could affect cefiderocol entry and susceptibility. Our homology and modeling analyses support a mechanistic basis for substrate competition or altered cefiderocol permeability in response to vitamin B12 exposure.

**Fig 3:**
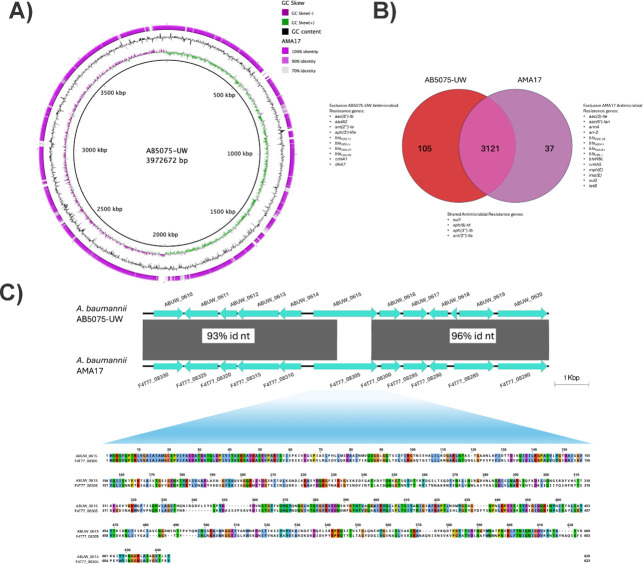
(**A**) Whole-genome comparison of *A. baumannii* strains AB5075-UW and AMA17 visualized with BRIG; from the innermost to outer rings: Ring 1, GC skew; Ring 2, GC content; Ring 3, BLAST similarity. (**B**) Venn diagram of gene content difference showing shared core genes (3,121 genes) and strain-specific genes (AB5075-UW: 105, AMA17: 37), reflecting clonal lineage differences (IC1/ST1 vs IC4/ST25). Antimicrobial resistance genes within the variable gene sets are annotated. (**C**) Schematic representation of the genomic context and amino acid sequence comparison of the most downregulated TonB-dependent vitamin B12 receptor genes (ABUW_0615 in AB5075 and F4T77_08305 in AMA17), highlighting protein-sequence differences and conserved genomic context.

### Global genomic and transcriptional analysis of *A. baumannii* AB5075 and AMA17

The genome-wide comparative analysis of both strains showed that although they share 3,026 genes, AB5075 and AMA17 harbor 105 and 37 unique genes, respectively (Fig. 3A and B). These differences most likely reflect their distinct clonal lineages (ST1 vs ST25) and evolutionary histories. Furthermore, the detection of approximately 60,727 single-nucleotide variants (SNVs) and 975 insertion/deletion events between the two genomes underscores the substantial genomic distance between these clones, highlighting the genetic heterogeneity that can exist within *A. baumannii* populations. In addition, a detailed genomic analysis of the ABUW_0615 and F4T77_08305 genes, which are homologs of the *E. coli* K-12 *btuB_3* gene and code for proteins related to B12 uptake in AB5075 and AMA17, respectively, revealed differences in the amino acid sequence of their cognate protein products (Fig. 3A and B). These differences, together with broader patterns of gene presence or absence and structural rearrangements (Fig. 3C and Table S4), likely contribute to the strain-specific transcriptional responses observed upon vitamin B12 exposure.

**Fig 4:**
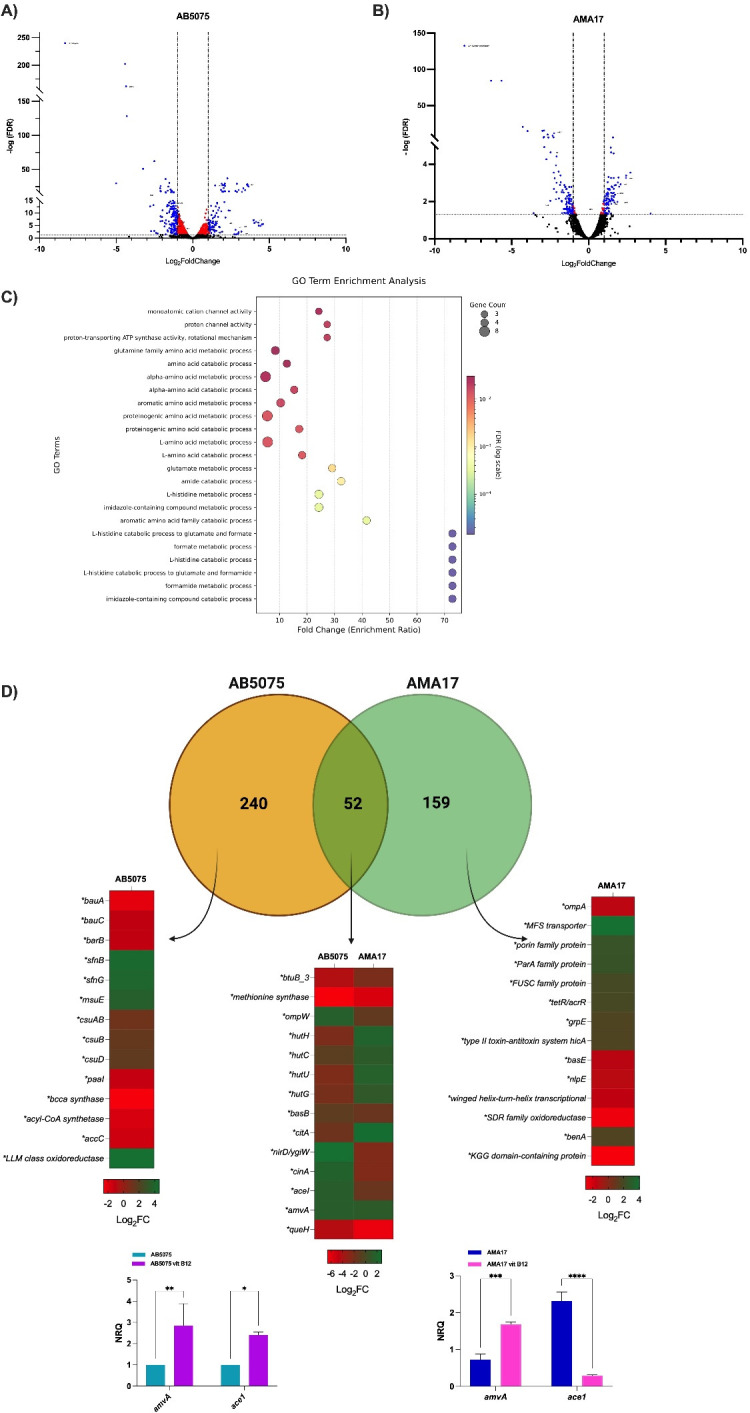
Volcano plots showing differentially expressed genes (DEGs) in *A. baumannii* strains AB5075 (**A**) and AMA17 (**B**) upon exposure to vitamin B12. Upregulated genes are highlighted in red; downregulated genes are highlighted in blue. (**C**) Gene Ontology (GO) functional categorization of DEGs identified in both strains, including pathways related to amino acid metabolism, iron acquisition, redox processes, membrane transport, and stress response. (**D**) Venn diagram illustrating the overlap of DEGs shared between AB5075 and AMA17, highlighting common transcriptional responses to vitamin B12 exposure.

The RNA-Seq analysis of AB5075 and AMA17 cells cultured in LB broth in the absence and presence of 100 mg/L vitamin B12 (methylcobalamin) showed that 292 out of 3,789 genes (7.71%) and 211 out of 3,902 genes (5.41%) were differentially expressed (DGE) in AB5075 and AMA17, respectively (Table S5). In AB5075, 115 and 177 genes were up- and downregulated, respectively, while 92 and 119 AMA17 genes were up- and downregulated, respectively, in response to vitamin B12 exposure (Fig. 4A and B). Overall, the differentially expressed genes (DEGs) were associated with a broad range of functions based on Gene Ontology analysis, including amino acid metabolism, iron acquisition, redox balance, membrane transport, and stress response pathways (Fig. 4C). Notably, 52 DEGs were shared between the two strains, indicating a partially conserved transcriptional response to vitamin B12 (Fig. 4D). Among the 52 shared DEGs, 38 of them displayed opposite regulation in response to vitamin B12, with several genes upregulated in one strain while downregulated in the other, that code for different functions, including oxidative stress defense and tolerance responses, histidine catabolism, chlorhexidine efflux pump, outer membrane proteins, and metabolic adaptation (Table S6; Fig. S4). These results suggest that the *A. baumannii* transcriptional response to vitamin B12 has a strain-specific component, likely associated with genomic differences, such as variations in gene content and synteny observed during the genome-wide analysis of these strains described above (Fig. 3A and Table S4). The most relevant differential responses to the presence of vitamin B12 related to the main focus of this work are reported below.

### Effects of vitamin B12 (methylcobalamin) on cellular functions

#### Metabolic functions

**Fig 5:**
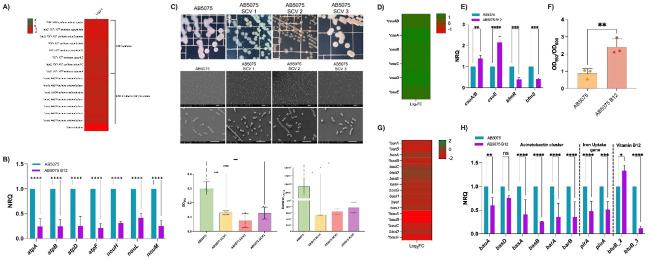
(**A**) Heatmap showing transcriptional changes in *A. baumannii* AB5075 respiratory chain genes upon vitamin B12 supplementation, including strong downregulation of ATP synthase and NADH-quinone oxidoreductase subunits. (**B**) Validation of RNA-seq results by qRT-PCR (calculated with qBASE) for selected genes (e.g., *atpB*, *atpF*, *atpD*, *atpF*, *nouH*, *nouL*, and *nouM*). (**C**) Morphological (top panels) and phenotypic characterization (bottom panels) of AB5075 and isogenic small-colony variants (SCVs). Representative colony morphologies of AB5075 and SCV1-SCV3 on CLED agar demonstrate reduced colony size in SCVs (top row). SEM images of AB5075 and SCVs showing altered cell morphology in the latter strains (middle columns, scale bars shown). Resazurin reduction assays measuring metabolic activity demonstrate significantly lower fluorescence intensity in SCVs, indicating reduced metabolic activity compared to AB5075 (bottom right graph). Statistical significance was determined by one-way ANOVA with post hoc testing. (**E and F**) Confirmation of the *csu* cluster upregulation by qRT-PCR (**E**) and increased biofilm formation determined by crystal violet assays (**F**). (**G**) Heatmap showing downregulation of genes coding for acinetobactin biosynthesis and transport functions. (**H**) qRT-PCR confirming the reduced expression of genes coding for acinetobactin-mediated iron acquisition functions, the TonB-dependent iron receptors *pirA* and *piuA*, and the predicted vitamin B12 receptors *btuB* and *btuB_3*. The data presented are the mean ± standard deviation (SD) of normalized relative quantities (NRQs) derived from transcript levels calculated using the qBASE method. Statistical significance (*P* < 0.05) was determined by one-way ANOVA followed by Tukey’s multiple-comparison test. *, *P* < 0.1; **, *P* < 0.01; ***, *P* < 0.001; ****, *P* < 0.0001. ns, not significant. Technical triplicates using three independent biological samples were used to collect experimental data. Error bars represent SDs.

Among the most significantly downregulated genes in both strains upon vitamin B12 exposure was the one coding for a DUF1852 domain-containing protein (ABUW_3198 in AB5075 and F4T77_07055 in AMA17), with log_2_ fold changes of −8.33 and −8.07, respectively (Fig. 4A and B and Table S5). This protein family has been previously linked to the activity of MesD-type folate-independent methionine synthases in aerobic bacteria, functioning as a partner in oxygen-dependent methyl group transfer (45). Its strong repression in both strains suggests a potential shift away from DUF1852-dependent methionine synthesis pathways in the presence of exogenous vitamin B12. Vitamin B12 supplementation also caused a marked metabolic reprogramming in both strains. Overall, there was a significant repression of genes coding for energy production functions (Table 1 and Table S5) and significantly decreased metabolic activity (Fig. 5C). This response would favor a lower-energy state that could reflect the emergence of SCVs with markedly reduced colony size and altered cellular morphology (Fig. 5C).

**TABLE 1:** Summary of selected differentially expressed genes in *A. baumannii* strain AB5075 upon vitamin B12 supplementation^*a*^

Gene	Name	Function	Log_2_ fold change	padj
ABUW_2336	SfnB family sulfur acquisition oxidoreductase	Environmental adaptation/sulfur acquisition	4.276333214	6.26E-07
ABUW_0178	sfnG, dimethyl sulfone monooxygenase SfnG	Environmental adaptation/sulfur acquisition	3.991941507	5.93E-08
ABUW_0179	msuE, FMN reductase	Environmental adaptation/sulfur acquisition	3.64029128	6.16E-26
ABUW_2414	Rhodanese-like domain-containing protein	Environmental adaptation/sulfur acquisition	3.57296659	3.39E-29
ABUW_2335	SfnB family sulfur acquisition oxidoreductase	Environmental adaptation/sulfur acquisition	3.548506553	8.51E-29
ABUW_2417	Cysteine desulfurase	Environmental adaptation/sulfur acquisition	3.306103815	3.65E-23
ABUW_3856	ssuC, aliphatic sulfonate ABC transporter permease	Environmental adaptation/sulfur acquisition	3.212320709	6.18E-18
ABUW_3857	ATP-binding cassette domain-containing protein	Environmental adaptation/sulfur acquisition	2.994088342	6.18E-18
ABUW_3854	Sulfonate ABC transporter substrate binding	Environmental adaptation/sulfur acquisition	2.885964706	5.38E-30
ABUW_3855	ssuD, FMNH2-dependent alkanesulfonate monooxygenase	Environmental adaptation/sulfur acquisition	2.813338191	1.68E-16
ABUW_2379	tauD, taurine dioxygenase	Environmental adaptation/sulfur acquisition	2.78189249	4.07E-19
ABUW_2380	tauC, taurine ABC transporter permease TauC	Environmental adaptation/sulfur acquisition	2.354355454	1.13E-18
ABUW_2946	cupin domain-containing protein	Environmental adaptation/sulfur acquisition	2.264561006	6.19E-38
ABUW_3853	Sulfonate ABC transporter substrate binding	Environmental adaptation/sulfur acquisition	2.10883105	9.48E-27
ABUW_1017	Sulfate ABC transporter ATP-binding protein	Environmental adaptation/sulfur acquisition	2.108111586	8.76E-19
ABUW_2943	Aspartate dehydrogenase	Environmental adaptation/sulfur acquisition	2.093017035	4.43E-30
ABUW_2381	ATP-binding cassette domain-containing protein	Environmental adaptation/sulfur acquisition	2.08279178	5.11E-19
ABUW_1570	Aliphatic sulfonate ABC transporter	Environmental adaptation/sulfur acquisition	2.013651046	9.38E-24
ABUW_2942	Aldehyde dehydrogenase	Environmental adaptation/sulfur acquisition	1.972647755	9.23E-28
ABUW_2420	Cysteine ABC transporter substrate binding	Environmental adaptation/sulfur acquisition	1.863931358	1.93E-11
ABUW_2382	tauA, taurine ABC transporter substrate binding	Environmental adaptation/sulfur acquisition	1.673926448	2.90E-16
ABUW_1569	ABC transporter permease	Environmental adaptation/sulfur acquisition	1.654003101	6.41E-12
ABUW_1016	CysB family HTH-type transcriptional regulator	Environmental adaptation/sulfur acquisition	1.598426734	1.45E-14
ABUW_0280	Sulfate ABC transporter substrate binding	Environmental adaptation/sulfur acquisition	1.267636585	1.02E-06
ABUW_2920	Arylsulfatase	Environmental adaptation/sulfur acquisition	1.156621368	3.05E-07
ABUW_1177	bauA, TonB-dependent ferric acinetobactin receptor	*Acinetobacter* siderophore system	−2.115835179	2.22E-19
ABUW_1174	bauC, ferric acinetobactin ABC transporter permease	*Acinetobacter* siderophore system	−1.1778157	1.30E-05
ABUW_1175	bauE, ferric acinetobactin ABC transporter ATP binding	*Acinetobacter* siderophore system	−1.628411402	9.68E-20
ABUW_1176	bauB, siderophore-binding periplasmic lipoprotein	*Acinetobacter* siderophore system	−2.100532991	1.52E-26
ABUW_1184	barA, acinetobactin export ABC transporter	*Acinetobacter* siderophore system	−1.012118423	4.19E-06
ABUW_1185	barB, acinetobactin export ABC transporter	*Acinetobacter* siderophore system	−1.202311434	4.19E-06
ABUW_1170	basB, acinetobactin non-ribosomal peptide synthetase	*Acinetobacter* siderophore system	−1.080635544	0.000897094
ABUW_1488	csuA, Csu fimbrial biogenesis protein CsuA	csu pili gene cluster	1.531892716	0.001071987
ABUW_1487	csuAB, Csu fimbrial major subunit CsuAB	csu pili gene cluster	1.124210917	0.00131199
ABUW_1489	csuB, Csu fimbrial biogenesis protein CsuB	csu pili gene cluster	1.412663268	0.0004707
ABUW_1490	csuC, Csu fimbrial biogenesis chaperone CsuC	csu pili gene cluster	1.391948896	3.08E-05
ABUW_1491	csuD, Csu fimbrial usher CsuD	csu pili gene cluster	1.566004659	1.13E-06
ABUW_1492	csuE, Csu fimbrial tip adhesin CsuE	csu pili gene cluster	1.212735633	4.90E-05
ABUW_3736	F0F1 ATP synthase subunit B	atp synthesis	−1.292499132	1.13E-14
ABUW_3737	atpE, F0F1 ATP synthase subunit C	atp synthesis	−1.102916814	4.61E-09
ABUW_3738	atpB, F0F1 ATP synthase subunit A	atp synthesis	−1.245453156	2.45E-10
ABUW_3735	F0F1 ATP synthase subunit delta	atp synthesis	−1.387703754	1.46E-14
ABUW_3734	F0F1 ATP synthase subunit alpha	atp synthesis	−1.437765183	2.70E-13
ABUW_3732	atpD, F0F1 ATP synthase subunit beta	atp synthesis	−1.6489912	5.63E-14
ABUW_3731	F0F1 ATP synthase subunit epsilon	atp synthesis	−1.677575657	1.82E-13
ABUW_3733	atpG, F0F1 ATP synthase subunit gamma	atp synthesis	−1.753343524	8.38E-17
ABUW_3167	nuoL, NADH-quinone oxidoreductase subunit L	Energy metabolism and redox	−1.107198047	1.72E-12
ABUW_3169	nuoJ, NADH-quinone oxidoreductase subunit J	Energy metabolism and redox	−1.154160198	2.92E-11
ABUW_3171	nuoH, NADH-quinone oxidoreductase subunit NuoH	Energy metabolism and redox	−1.199129502	7.15E-16
ABUW_3166	nuoM, NADH-quinone oxidoreductase subunit M	Energy metabolism and redox	−1.235543878	3.59E-12
ABUW_3168	nuoK, NADH-quinone oxidoreductase subunit NuoK	Energy metabolism and redox	−1.268195967	4.72E-11
ABUW_3165	nuoN, NADH-quinone oxidoreductase subunit NuoN	Energy metabolism and redox	−1.330875591	2.18E-13
ABUW_3170	nuoI, NADH-quinone oxidoreductase subunit NuoI	Energy metabolism and redox	−1.417797477	1.72E-17
ABUW_0958	electron transfer flavoprotein subunit beta/FixA	Energy metabolism and redox	−1.067842283	4.83E-07
ABUW_2237	NAD(*P*)/FAD-dependent oxidoreductase	Energy metabolism and redox	−1.155467112	0.00011107
ABUW_0876	sucD, succinate-CoA ligase subunit alpha	Energy metabolism and redox	−1.177878219	1.07E-14
ABUW_3313	NAD(*P*) transhydrogenase subunit alpha	Energy metabolism and redox	−1.231865183	7.42E-08
ABUW_3312	NAD(*P*)(+) transhydrogenase (Re/Si specific)	Energy metabolism and redox	−1.250217635	2.41E-09
ABUW_3314	Re/Si-specific NAD(*P*)(+) transhydrogenase	Energy metabolism and redox	−1.300379036	2.08E-09
ABUW_3196	Flavin reductase	Energy metabolism and redox	−2.518052231	4.97E-63
ABUW_3198	DUF1852 domain-containing protein	Energy metabolism and redox	−8.331114036	7.67E-241

^*a*^ The table includes genes related to energy metabolism (e.g., ATP synthase complex and NADH-quinone oxidoreductase subunits), sulfur metabolism and transport (sfnG, sfnB, msuE, tauD, ssuD, and ssuC), biofilm formation (csu pili gene cluster), and iron acquisition systems (acinetobactin biosynthetic genes and TonB-dependent receptors). Fold changes are shown as log_2_ values relative to untreated controls.

**Fig 6:**
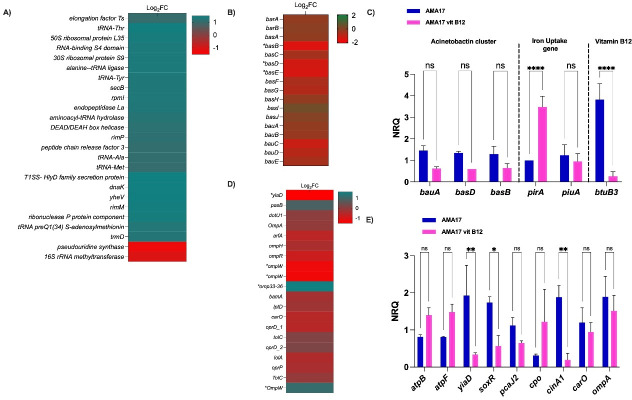
(**A**) Heatmap showing upregulation of translation-related genes in *A. baumannii* AMA17 after vitamin B12 supplementation, including ribosomal proteins, elongation factors, tRNA synthetases, and RNA modification enzymes. (**B**) Downregulation of genes coding for acinetobactin biosynthesis and transport functions following vitamin B12 exposure. (**C**) qRT-PCR confirming the reduced expression of genes coding for acinetobactin-mediated iron acquisition functions, the TonB-dependent iron receptors *pirA* and *piuA*, and the predicted vitamin B12 receptor *btuB_3*. (**D**) Heatmap illustrating widespread downregulation of outer membrane porin genes, with several displaying strong repression in response to vitamin B12. (**E**) qRT-PCR confirming the differential expression of genes related to energy metabolism (*atpB*, *atpF*, and *amvA*), compound transport (*yiaD* and *cpo*), oxidative stress response (*soxR*), aromatic compound metabolism (*pcaJ2*), and DNA repair (*cinA1*). The qRT-PCR data are presented as mean ± standard deviation (SD) of normalized relative quantities (NRQs), calculated from transcript levels using the qBASE method. Statistical significance (*P* < 0.05) was determined by one-way ANOVA followed by Tukey’s multiple-comparison test. *, *P* < 0.1; **, *P* < 0.01; ***, *P* < 0.001; ****, *P* < 0.0001. ns, not significant. Technical triplicates using three independent biological samples were used to collect experimental data. Error bars represent SDs.

In contrast to AB5075, exposure of AMA17 to vitamin B12 induced the upregulation of genes coding for the translation machinery and protein biogenesis, indicating an increased translational capacity response (Table 2 and Fig. 6A). This response indicates that AMA17 may adapt to vitamin B12 exposure by maximizing protein production and import of essential substrates to support its metabolism under stress or altered environmental conditions.

**TABLE 2:** Selected differentially expressed genes in *A. baumannii* strain AMA17 following vitamin B12 supplementation^*a*^

Gene	Name	Function	Log_2_ fold change	padj
F4T77_00520	Elongation factor Ts	Translation and protein biogenesis	1.01093468	0.008100626
F4T77_11615	tRNA-Thr	Translation and protein biogenesis	1.454695864	0.04472478
F4T77_11025	50S ribosomal protein L35	Translation and protein biogenesis	1.382263922	0.002304368
F4T77_17840	RNA-binding S4 domain-containing protein	Translation and protein biogenesis	1.328976107	0.035755415
F4T77_18775	30S ribosomal protein S9	Translation and protein biogenesis	1.328238139	0.00210376
F4T77_08590	Alanine-tRNA ligase	Translation and protein biogenesis	1.324881271	0.002304368
F4T77_11625	tRNA-Tyr	Translation and protein biogenesis	1.293023849	0.01060046
F4T77_01900	Protein-export chaperone SecB	Translation and protein biogenesis	1.288340049	0.01500704
F4T77_11020	rpmI, 50S ribosomal protein L35	Translation and protein biogenesis	1.280159189	0.00393599
F4T77_06050	Endopeptidase La	Translation and protein biogenesis	1.263927853	0.00711625
F4T77_07540	Aminoacyl-tRNA hydrolase	Translation and protein biogenesis	1.205578143	0.00400906
F4T77_04970	DEAD/DEAH box helicase	Translation and protein biogenesis	1.138802189	0.00686292
F4T77_02785	Ribosome maturation factor RimP	Translation and protein biogenesis	1.130323182	0.0128096
F4T77_18665	Peptide chain release factor 3	Translation and protein biogenesis	1.059636936	0.00900307
F4T77_20355	tRNA-Ala	Translation and protein biogenesis	1.059444114	0.03106752
F4T77_02790	tRNA-Met	Translation and protein biogenesis	1.030215868	0.04248775
F4T77_02870	T1SS- HlyD family secretion protein	Translation and protein biogenesis	1.485315347	0.00509345
F4T77_16880	Molecular chaperone DnaK	Translation and protein biogenesis	1.471953746	0.013437858
F4T77_05270	YheV family putative zinc ribbon protein	Translation and protein biogenesis	1.460900317	0.003385741
F4T77_04675	Ribosome maturation factor RimM	Translation and protein biogenesis	1.438462818	0.000012807
F4T77_16755	Ribonuclease P protein component	Translation and protein biogenesis	1.403242445	0.009204377
F4T77_18995	tRNA preQ1(34) S-adenosylmethionin	Translation and protein biogenesis	1.259347926	0.02267644
F4T77_04680	tRNA [guanosine(37)-N1]-methyltransferase TrmD	Translation and protein biogenesis	1.238069081	0.00091925
F4T77_16135	Pseudouridine synthase	Translation and protein biogenesis	−1.290019759	0.00790266
F4T77_00585	16S rRNA [uracil(1498)-N(3)]-methyltransferase	Translation and protein biogenesis	−1.060180299	0.04173287
F4T77_04910	Putative DNA modification/repair radical SA	DNA chemical modifications/replication	1.535528546	0.008300274
F4T77_12295	Chaperonin GroEL	Cell adaptation and stress response/protein folding and chaperones	1.485622008	0.00681922
F4T77_18770	Glutathione S-transferase N-terminal domain	Cell detoxification/protects cells from oxidative stress	1.457699101	0.00054746
F4T77_02980	Molecular chaperone HtpG	Protein quality control/stress response: heat shock proteins	1.343682554	0.00922443
F4T77_08535	ATP-dependent chaperone ClpB	Cell adaptation: protein quality control/heat shock and stress response	1.333814511	0.007675671
F4T77_10125	Molecular chaperone DnaJ	Cell adaptation/protein quality control/stress response–molecular chaperones	1.304553852	0.02335136
F4T77_16750	Chromosomal replication initiator protein DnaA	Cell adaptation/DNA replication	1.256515135	0.00518164
F4T77_00340	NirD/YgiW/YdeI family stress tolerance protein	Stress tolerance/cellular adaptation	−2.47618744	0.000022789
F4T77_06560	CinA family protein	Stress tolerance/adaptive processes (natural competition, repair, NAD^+^)	−2.49269497	0.00922443
F4T77_05940	Copper resistance protein NlpE	Stress response membrane lipoprotein, possibly involved in metal resistance	−1.429705586	0.010138863
F4T77_08240	Peroxiredoxin	Stress response/detoxification	−1.272327071	0.029490576
F4T77_02640	Chaperone modulator CbpM	Cellular adaptation and stress response/protein folding and refolding	−1.143917377	0.00940867
F4T77_04765	Superoxide dismutase family protein	Cellular adaptation and stress response/bacterial antioxidant defense	−1.111417705	0.0279096
F4T77_13150	RsiV family protein	Cellular adaptation and stress response	−1.090519674	0.02121219

^*a*^ The table includes genes involved in translation and protein biosynthesis (ribosomal proteins, elongation factors, tRNA synthetases, and RNA modification enzymes), membrane transporters (major facilitator superfamily, ABC transporters, and permeases), and stress response pathways (heat shock proteins GroEL, DnaA, DnaJ, HtpG, ClpB, and oxidative stress-related proteins). Fold changes are presented as log_2_ values relative to untreated controls.

Both AB5075 and AMA17 showed downregulation of genes coding for phenylacetic acid catabolism in the presence of vitamin B12 (Fig. 6A), a marked contrast to the different response of their *hut* operons involved in histidine catabolism (Fig. 6B). This divergent regulation suggests strain-specific metabolic patterns, possibly linked to differences in nitrogen assimilation or regulatory functions.

#### Stress response functions

Another observed difference in AMA17 transcriptional response compared to AB5075 was the differential expression of genes associated with stress responses, detoxification, and cellular adaptation (Table 2 and Fig. S5D). Among these DGEs are those involved in protein quality control and refolding, such as *groEL*, *dnaA*, *dnaJ*, *htpG*, and *clpB*, which were upregulated when cells were exposed to vitamin B12 (Table 2 and Fig. S5D). In contrast, seven stress tolerance genes, including *nirD* and *cinA*, were significantly downregulated under the same experimental condition (Table 2 and Fig. S5D).

#### Efflux and membrane transport functions

The analysis of genes coding for efflux-pump functions, some of which are associated with antibiotic resistance, showed transcriptional differences between the two strains when exposed to vitamin B12. In AB5075, several components of the AdeABC multidrug efflux system (*adeA*, *adeB*, and *adeC*) were downregulated, along with *adeIJK*. At the same time, moderate upregulation was observed for *adeF*, *adeG*, and *adeH* of the AdeFGH system. The *emrA* gene encoding a multidrug resistance efflux pump was notably downregulated (Fig. S6C and Table S5). In contrast, the AMA17 *adeI*, *adeJ*, *emrA*/*ermK*, *emrA*, and *emrB* genes were upregulated (Fig. S6C and Table S5). Interestingly, the expression of the *baeR*/*baeS* two-component regulatory system, which controls the expression of genes coding for efflux functions, was mildly upregulated in AB5075 but downregulated or unchanged in AMA17 (Fig. S6C and Table S4). Another distinct feature of AMA17 was the marked downregulation of outer membrane porin genes, with 15 out of 20 of them showing reduced expression and 3 of them exhibiting strong repression (Fig. 6E and Table S5). In contrast, *amvA*, coding for a member of the major facilitator superfamily protein associated with multidrug efflux and metabolic stress adaptation, was upregulated in both strains cultured in the presence of vitamin B12 (Fig. 4D).

#### Biofilm formation functions

Another distinct feature observed in AB5075’s response to vitamin B12 was the significant upregulation of the entire *csu* pili gene cluster (Fig. 5D), data that were confirmed by RT-qPCR and the increased formation of biofilms on plastic tubes (Fig. 5E and F). This response could contribute to the persistence of this strain during its interaction with the host since the products of the *csu* operon play a critical role in surface attachment and biofilm formation (46, 47). Unlike AB5075, AMA17 displayed downregulation of the *csu* pili gene cluster based on RNA-seq data; however, these genes did not reach statistical significance for differential expression (Fig. S7 and Table S5). This result was further confirmed by RT-qPCR and biofilm formation assays, both of which also showed no significant differences upon vitamin B12 supplementation (Fig. S7), suggesting that *csu* expression and biofilm formation capacity were not markedly affected by vitamin B12 in AMA17.

#### TonB-dependent transport functions

The RNA-Seq data also showed that the presence of vitamin B12 affected the expression of genes coding for active iron acquisition systems. The expression of the *bauA*, *bauB*, and *bauE* genes, which code for acinetobactin transport, was strongly downregulated in both strains (Fig. 5G and H, 6B and C). Furthermore, both strains showed downregulation of the *basB* acinetobactin biosynthesis gene when cultured under the same conditions (Fig. 4D). The data also showed that vitamin B12 downregulated the expression of additional iron-uptake system receptors (e.g., *pirA* and *piuA*) and several uncharacterized TonB-dependent receptors (Fig. 5H and Fig. S5A and 5C). This response to vitamin B12 could limit the cellular entry of siderophore antibiotic conjugates, such as cefiderocol, leading to cefiderocol resistance.

In summary, the transcriptomic analysis revealed that vitamin B12 induces a unique global adaptive cellular and functional response that would contribute to the overall ability of *A. baumannii* to persist and resist the presence of cefiderocol in the extracellular medium.

### Cefiderocol MIC increases in CR *Acinetobacter* spp. and *A. baumannii* model strains under vitamin B12 exposure

To determine whether vitamin B12 exposure influences cefiderocol susceptibility in carbapenem-resistant *Acinetobacter* (CRA) and commonly used *A. baumannii* laboratory strains, MIC values were determined using iron-depleted cation-adjusted Mueller–Hinton broth (ID-CAMHB) in the absence and presence of 100 mg/L vitamin B12 (methylcobalamin). A total of 15 *Acinetobacter* strains, including reference strains such as AB5075, AYE, ATCC 17978, ATCC 19606, and clinical isolates representing distinct carbapenemase genotypes and sequence types, were tested (Table 3).

**TABLE 3:** MICs of FDC in the presence or absence of vitamin B12 (methylcobalamin^*a*^) supplementation in carbapenem-resistant and carbapenem-susceptible *Acinetobacter* clinical and model strains^*b*^

Strain	Characteristic	ST^*c*^	Carbapenemase	FDC MIC (mg/L)	
				ID-CAMHB	ID-CAMHB + B12^*a*^ (100 mg/L)
AMA5 (*A. baumannii*)	CRAB	25	*bla*_NDM-1_	32	64
AMA17 (*A. baumannii*)	CRAB	25	*bla*_NDM-1_	32	>128
AMA32 (*A. junii*)	CRAJ	NA	*bla*_NDM-1_	1	4
AMA2 (*A. baumannii*)	CRAB	25	*bla*_NDM-1_	1	4
AMA22 (*A. baumannii*)	CRAB	25	*bla*_NDM-1_	1	8
AMA122 (*A. baumannii*)	CRAB	25	*bla*_NDM-1_	8	8–16
AB27850 (*A. baumannii*)	CRAB	NA	*bla*_OXA-23_	0.5	0.5
AB5075^*d*^ (*A. baumannii*)	CRAB	1	*bla*_OXA-23_	0.5–1.0	8
AR Bank #0056 (*A. baumannii*)	CRAB	2	*bla*_OXA-23_	0.5–2.0	16
AR Bank #0033^*d*^ (*A. baumannii*)	CRAB	85	*bla*_NDM-1_	4–16	64
AB0057^*d*^ (*A. baumannii*)	CRAB	1	*bla*_OXA-23_	1	64
AYE^*d*^ (*A. baumannii*)	CRAB	1	*bla*_OXA-23_	32	>128
A118^*d*^ (*A. baumannii*)	Susceptible	98	None	0.5	0.5
ATCC 19606^*d*^ (*A. baumannii*)	Susceptible	52	None	0.125	0.25
ATCC 17978^*d*^ (*A. baumannii*)	Susceptible	437	None	0.05	0.2

^*a*^ Sigma-Aldrich (C_63_H_91_CoN_13_O_14_P).

^*b*^ CRAB, carbapenem-resistant *Acinetobacter baumannii*; FDC, cefiderocol; MIC, minimum inhibitory concentration; NA, not available.

^*c*^ Pasteur scheme.

^*d*^ Model strains.

We observed that vitamin B12 supplementation increased cefiderocol MICs in 10 of the 12 CRA tested strains (Table 3). Among *bla*_NDM-1_ positive isolates, the cefiderocol MICs increased between one and eight twofold dilutions; for example, AMA2 and AMA22 rose from 1 to 4–8 mg/L, and AMA17 showed an increase from 32 to >128 mg/L (Table 3). The reference strain AB5075, carrying *bla*_OXA-23_, exhibited a fourfold dilution increase (from 0.5 to 1.0–8.0 mg/L). The carbapenem-susceptible model strains, such as A118 and ATCC 19606, exhibited no changes or minimal increases, while ATCC 17978 exhibited a twofold dilution increase in MIC, remaining within the susceptible range (Table 3).

To determine whether the observed effect on cefiderocol susceptibility was specific to a particular form of vitamin B12, we tested the derivative cyanocobalamin, purchased from two different vendors, against selected CRA species isolates and the *E. coli* K12 strain coding for *bla*_NDM-5_ (Table 4). As shown in Table 4, the presence of methylcobalamin or cyanocobalamin, which was obtained from two different sources, resulted in the same fourfold dilution increase in cefiderocol MIC, from 0.25 to 4.0 mg/L, for the *E. coli* K12 *bla*_NDM-5_ strain. In contrast, *A. baumannii* strains demonstrated a different response, with cefiderocol MIC increases observed primarily in the presence of methylcobalamin. For the CRA tested strains, MICs remained unchanged or minimally affected with both cyanocobalamin brands tested. These findings suggest that methylcobalamin uniquely potentiates cefiderocol resistance in certain *A. baumannii* strains, while all vitamin B12 forms can induce resistance in *E. coli* K12 NDM-5. In addition, different brands of methylcobalamin were tested, showing the same results as reported with the previous brand (data not shown). In addition, to assess whether this phenomenon also occurs in other gram-negative pathogens, we determined cefiderocol MICs in the presence of methylcobalamin (100 mg/L) for *K. pneumoniae* (Kp27278, Kp01, KpJAF1, KpZBE2, and KPZCA8), *E. coli* (Ec7499, EcZBB4, and EcZAU4), and *Pseudomonas aeruginosa* (PAE319, PAE27829, and PAE27875). Cefiderocol MICs increased by up to a threefold dilution in *K. pneumoniae*, up to a twofold dilution in *E. coli*, and up to a fourfold dilution in *P. aeruginosa* (Table S7). These findings indicate that vitamin B12 can modulate cefiderocol susceptibility across multiple gram-negative species, extending its effect beyond *A. baumannii*.

**TABLE 4:** Cefiderocol MIC in the presence of two different forms or brands of vitamin B12 against *E. coli* and *A. baumannii* strains

Strain	Cefiderocol MIC mg/L (broth microdilution)			
	ID-CAMHB	ID-CAMHB + methylcobalamin^*b*^ (100 mg/L)	ID-CAMHB + cyanocobalamin^*a*^ (100 mg/L)	ID-CAMHB + cyanocobalamin^*b*^ (100 mg/L)
EC K12 NDM-5	0.25	4	4	4
AMA 2	1	4	1	2
AMA 5	32	64	32	32
AMA 17	32	>128	64	64
AMA 32	1	4	1	2

^*a*^ Supelco brand.

^*b*^ Sigma-Aldrich brand.

**Fig 7:**
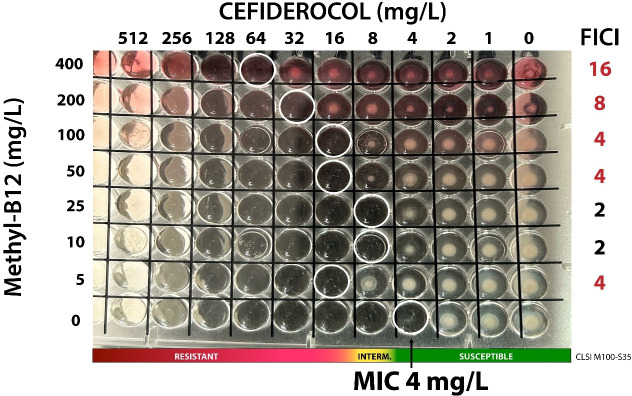
Checkerboard assay evaluating the impact of methylcobalamin (0–400 mg/L) on cefiderocol susceptibility in the *A. baumannii* reference strain (CDC AR Bank #0033). Heatmaps display cefiderocol MIC shifts in the presence of increasing methylcobalamin concentrations. The fractional inhibitory concentration index (FICI) was calculated to determine antagonism (FICI >4.0). Bar plots show cefiderocol MICs of colonies recovered after exposure to high methylcobalamin concentrations (>100 mg/L) and serially passaged in drug-free medium, revealing persistent MIC increases relative to baseline.

To confirm the critical concentrations of methylcobalamin that antagonize cefiderocol, a checkerboard assay was performed using concentrations ranging from 0.25 to 256.0 mg/L, and methylcobalamin from 0 to 400 mg/L. Two *A. baumannii* reference strains with distinct genetic backgrounds were tested: CDC AR Bank #0033, which carries *bla*_NDM-1_, *bla*_OXA-94_, *sul_2_*, ABAF, *ant(3″)-Ia*, and *ble*_MBL_ and has a reported cefiderocol MIC of 4–16 mg/L; and CDC AR Bank #0056, which carries *bla*_OXA-23_ and *bla*_OXA-66_, with a reported cefiderocol MIC of 0.5–2.0 mg/L. The fractional inhibitory concentration index (FICI) was calculated, with antagonism defined as a FICI of >4. Methylcobalamin concentrations of >25 mg/L consistently produced antagonistic effects, resulting in a two- to four-fold increase in cefiderocol MICs relative to baseline values (Fig. 7). To assess the stability of this effect, survivors grown in the presence of >16 mg/L cefiderocol and 100–400 mg/L methylcobalamin were subcultured for 10 successive passages on trypticase soy agar without methylcobalamin. Reevaluation of cefiderocol susceptibility revealed a persistent fourfold MIC increase in both strains (64 mg/L for AR Bank #0033 and 16 mg/L for AR Bank #0056), indicating that a single exposure to these methylcobalamin concentrations was sufficient to induce irreversible phenotypic changes affecting cefiderocol susceptibility.

Time-kill assays using at least 4× MIC of cefiderocol were performed to evaluate the impact of methylcobalamin on bactericidal kinetics for the AB5075 model strain and five other CR strains, including the two CRAB reference CDC bank strains (AR Bank #0033 and AR Bank #0056) and three additional Gram-negative CR bacilli strains: *E. coli* (Ec7499), *K. pneumoniae* (Kp01), and *P. aeruginosa* (PAE319) (Fig. S8). At cefiderocol 4× MIC, two distinct response patterns were observed. In AR Bank #0033, Ec7499, and Kp01, the antibiotic showed clear bactericidal activity, reducing viable counts to or near the limit of quantification by 24 h. In contrast, AB5075, AR Bank #0056, and PAE319 exhibited a more bacteriostatic profile: in AB5075 and AR Bank #0056, viability decreased by 1–2 log_10_ during the first 6 h and then stabilized at ~10⁵ CFU/mL by 24 h, while in PAE319, counts increased by ~0.5 log_10_ at 2 h and subsequently returned to baseline, also remaining around 10⁵ CFU/mL at the end of the assay. However, the addition of methylcobalamin (100 mg/L) consistently impaired cefiderocol activity. In all strains except PAE319, the early killing kinetics observed with 4× MIC cefiderocol were initially preserved during the first 6 h, but this effect was followed by regrowth toward 24 h. Regrowth was most pronounced in AB5075 and Ec7499, where viable counts returned to levels comparable to the ID-CAMHB control (Fig. S8A through D). The remaining strains (AR Bank #0033, AR Bank #0056, and Kp01) also displayed regrowth (Fig. S8B and C, E). In PAE319, no detectable cefiderocol killing activity was observed in the presence of methylcobalamin; its growth kinetics closely mirrored the ID-CAMHB control throughout the assay, indicating that methylcobalamin fully abolished the already minimal activity observed with cefiderocol alone (Fig. S8F). Notably, survivors isolated after regrowth remained resistant upon subsequent passages in antibiotic-free medium, confirming the selection of stably resistant subpopulations. These findings further support the role of methylcobalamin in impairing cefiderocol efficacy and promoting the emergence of subpopulations with reduced susceptibility.

Based on the time-kill assay results, where bacterial regrowth occurred upon exposure to methylcobalamin and cefiderocol, a randomly selected survivor of the CDC bank strain (AR Bank #0033) at 24 h was sent for WGS and analysis. Comparative genomic analysis between the AR Bank #0033 wild-type strain and the randomly selected mutant identified a total of 12 mutations, 3 of which were in coding regions (Table S1). Among these, one mutation corresponded to an 18-nt in-frame deletion in *pirA*, which encodes a TonB-dependent outer membrane receptor previously associated with cefiderocol resistance (48, 49) (Table S1). In addition, we detected a mutation in *panB* (Y23*), encoding 3-methyl-2-oxobutanoate hydroxymethyltransferase, an enzyme that catalyzes the transfer of a CH_2_OH group from 5,10-methylene-THF to α-ketoisovalerate to form ketopantoate, the first committed step in pantothenate biosynthesis (50) (Table S1). Given the central role of *panB* in the pantothenate/CoA pathway and the association of *panB* defects with pantothenate auxotrophy, reduced CoA levels, and SCV phenotypes in other species (51), the *panB* mutation observed in the AR Bank #0033 mutant provides a plausible explanation for its SCV-like growth phenotype.

To further explore the dose-dependent impact of methylcobalamin on cefiderocol susceptibility, we expanded the analysis by testing two concentrations (40 and 100 mg/L) using selected representative strains (Table 5). A concentration-dependent effect was confirmed. In *E. coli* K12 NDM-5, the cefiderocol MIC increased from 0.25 to 2.0 mg/L with 40 mg/L methylcobalamin and to 4 mg/L with 100 mg/L methylcobalamin (Table 5). Similarly, in AMA2 and AMA32, MICs increased by one- and twofold dilutions, while AMA5 and AMA17 reached or exceeded an MIC of >128 mg/L (Table 5). Since we did not observe notable changes in cefiderocol MICs for the susceptible ATCC strains at 100 mg/L vitamin B12, we tested a higher concentration (200 mg/L) to evaluate a potential dose-dependent effect. At this increased concentration, we observed a threefold dilution increase in cefiderocol MICs, confirming that vitamin B12 may impact cefiderocol susceptibility in a concentration-dependent manner, even in susceptible strains (Table S8).

**TABLE 5:** Cefiderocol MICs in the presence of increasing concentrations of methylcobalamin in selected *E. coli* and *A. baumannii* strains

Strain	Cefiderocol MIC mg/L (broth microdilution)		
	ID-CAMHB	ID-CAMHB + methylcobalamin^*a*^ (400 mg/L)	ID-CAMHB + methylcobalamin^*a*^ (100 mg/L)
EC K12 NDM-5	0.25	2	4
AMA 2	1	2	4
AMA 5	32	>128	64
AMA 17	32	>128	>128
AMA 32	1	2	4

^*a*^ Sigma-Aldrich (C_63_H_91_CoN_13_O_14_P).

Lastly, to assess if vitamin B12 interacts with host-derived environments, we next evaluated the effect of vitamin B12 (methylcobalamin) supplementation on cefiderocol susceptibility under conditions mimicking human physiological fluids. The cefiderocol MICs were measured in ID-CAMHB supplemented with 4% or 10% HPF, or 3.5% human serum albumin (HSA) in the presence or absence of methylcobalamin (100 mg/L). These combinations revealed increases in cefiderocol MICs in the tested strains (Table S9). In AB5075, the MIC increased from 0.5 to 1.0 mg/L in ID-CAMHB to 128 mg/L in 10% HPF + methylcobalamin. Similarly, AMA17 and AMA32 reached or exceeded 512 mg/L under vitamin B12 and host fluid conditions (Table S9). These findings support and agree with previous reports demonstrating that host fluids such as HPF and HSA can modulate antibiotic susceptibility in *A. baumannii* and influence transcriptomic response (31, 52–55). Moreover, this effect aligns with our observation in *E. coli* K12 NDM-5, where an HPF-induced cefiderocol-resistance phenotype was found. Taken together, these results suggest that vitamin B12 acts synergistically with host environmental signals to enhance cefiderocol resistance, potentially by altering iron availability, outer membrane permeability, or siderophore-transport competition.

Taken together, these results indicate that vitamin B12 may modulate cefiderocol susceptibility in a strain- and resistance genotype-dependent manner, with methylcobalamin producing the most pronounced effects, particularly in *A. baumannii*. In contrast, all vitamin B12 forms tested enhanced resistance in *E. coli* K12 NDM-5 but not in the *A. baumannii* tested strains, indicating a broader impact on bacterial physiology. The observed concentration-dependent increases in MICs, along with the additive effects of vitamin B12 in the presence of host-derived fluids such as HPF, further support the hypothesis that vitamin B12 interferes with cefiderocol uptake, iron homeostasis, and stress response pathways associated with metal acquisition and adaptation to host environments.

### Confirmation of vitamin B12 selection for SCVs

**Fig 8:**
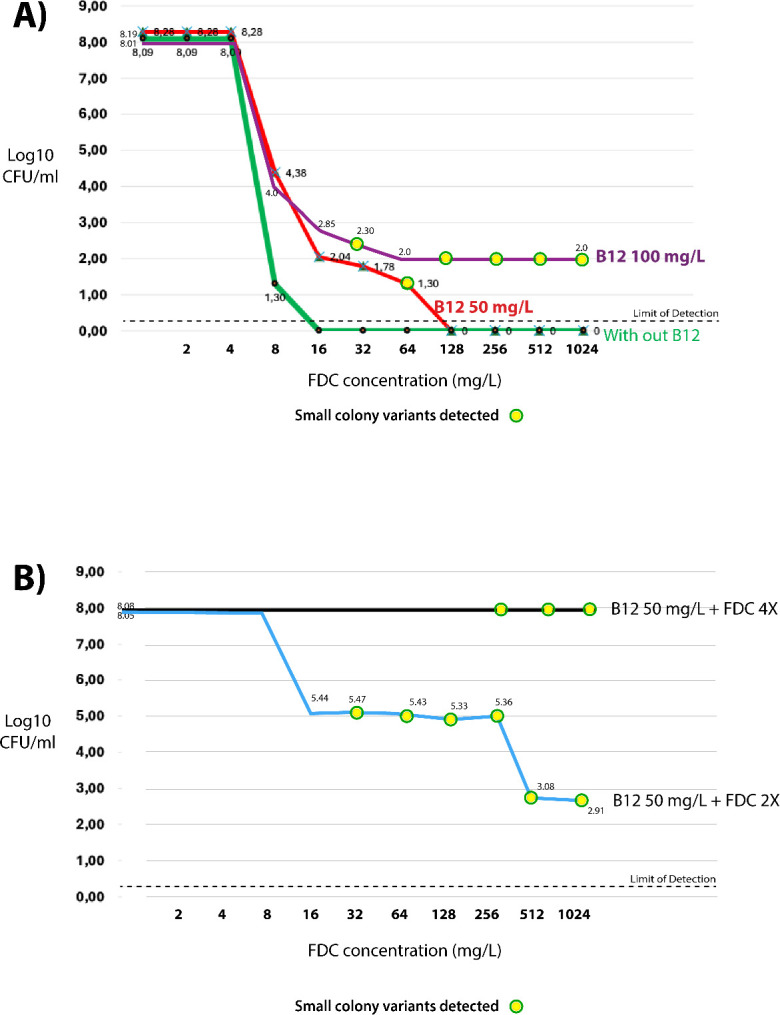
(**A**) Population analysis profile (PAP) of *A. baumannii* CDC AR Bank #0033 exposed to increasing concentrations of cefiderocol in the presence or absence of B12 (methylcobalamin 50 and 100 mg/L). Curves illustrate the emergence of heteroresistant subpopulations and small-colony variants at higher vitamin B12 concentrations. (**B**) PAP of surviving colonies recovered from time-kill assays after exposure to FDC and 50 mg/L vitamin B12, showing changes in population structure. Colonies exposed to 4× MIC of FDC developed a uniformly resistant phenotype, while those exposed to 2× MIC exhibited dose-dependent heteroresistance.

As observed in the transcriptomic data, there is a clear shift in the expression of metabolic genes from slow to metabolic dormancy, suggesting the occurrence of SCVs (Fig. 5C). As mentioned above, this phenomenon has not been previously reported in *A. baumannii*, which prompted us to perform a population analysis profile (PAP) using the model strain *A. baumannii* CDC AR Bank #0033. This strain was exposed to increasing concentrations of cefiderocol in the presence and absence of two selected concentrations of vitamin B12 (methylcobalamin). PAP analysis revealed that exposure to vitamin B12 progressively reduced the bactericidal activity of cefiderocol, with the area under the curve increasing from 147,647 in the absence of vitamin B12 to 237,954 and 544,151 in the presence of 50 and 100 mg/L vitamin B12, respectively (*P* < 0.001). This shift was associated with the emergence of heteroresistant subpopulations. Notably, a sustained survival plateau was observed at cefiderocol concentrations of ≥32 mg/L in the presence of 100 mg/L vitamin B12, suggesting the development of stable resistance. SCVs emerged only after exposure to vitamin B12 at high cefiderocol concentrations, becoming more abundant at the higher vitamin B12 dose and persisting up to 1,024 mg/L of cefiderocol. These findings support the hypothesis that vitamin B12 promotes the selection or induction of SCVs with enhanced survival capacity, even at high levels of cefiderocol (Fig. 8A). Surviving strains selected from the time-kill assay after exposure to vitamin B12 were reevaluated using PAP to assess changes in population structure. The results confirmed that exposure to high cefiderocol concentrations (4× MIC) and 50 mg/L of vitamin B12 led to the emergence of a uniformly resistant population, indicative of homogeneous and high-level resistance. In contrast, survivors exposed to 2× MIC and 50 mg/L of vitamin B12 also displayed a dose-dependent heteroresistance (Fig. 8B). Notably, this shift in population dynamics was observed after 6 h of exposure to vitamin B12, highlighting the rapid and potentiating effect of B12 on cefiderocol resistance development. Thus, populations previously exposed to vitamin B12 more readily progress to high-level, homogeneous cefiderocol resistance upon subsequent exposure cycles, a scenario that could markedly accelerate the emergence of stably resistant strains in patients repeatedly receiving vitamin B12 within this selection window.

## DISCUSSION

Antibiotic resistance to last-resort agents such as cefiderocol is an emerging concern, with numerous mechanisms and molecular mechanisms still poorly characterized or entirely unknown. In *A. baumannii*, a highly multidrug-resistant pathogen, antibiotic resistance is even more complex due to its recognized genetic variability and strain-specific response factors that limit the ability to draw generalizable conclusions for guiding effective therapy. Only a limited number of antibiotics remain effective against highly resistant *A. baumannii* strains, with cefiderocol being one of the few remaining options. However, growing concerns have emerged as multiple reports document an increasing number of cefiderocol-resistant isolates and clinical failures in the treatment of *A. baumannii* infections.

Recent observations reveal that *A. baumannii* can sense and respond to various human fluids and host-derived proteins, triggering transcriptional changes that include increased cefiderocol MICs. While studying an *E. coli* harboring NDM-5 under host-mimicking conditions, we unexpectedly observed the emergence of a cefiderocol-resistant colony with a mutation on a putative vitamin B12 receptor gene (*btuB_3*). This finding led us to hypothesize that, apart from the influence of human fluids and host proteins, human supplements like vitamin B12 may also modulate antibiotic resistance phenotypes. This observation prompted the rationale for the present study, which aims to investigate the impact of vitamin B12 exposure on *A. baumannii* gene regulation and antibiotic susceptibility. Few studies have addressed the role of vitamin B12 in bacterial physiology. Groon et al. showed that vitamin B12 affects *V. campbellii* transcriptional response, modulating the expression of genes involved in methionine biosynthesis, central metabolism, and genes associated with motility and virulence (36). In addition, another recent work in *Mycobacterium tuberculosis*, a human pathogen that cannot synthesize vitamin B12, showed that exogenous vitamin B12 uptake is needed for virulence and growth (56).

In addition, the substrate specificity of many TBDRs remains poorly understood, leaving key aspects of their biological roles and transport functions uncharacterized. The structural and sequence-based analyses presented here provide evidence that TBDRs in *A. baumannii* and other gram-negative bacteria exhibit a degree of substrate promiscuity, with overlapping specificity for structurally distinct molecules such as vitamin B12 and siderophore-conjugated antibiotics like cefiderocol. This finding has important implications for understanding non-canonical cefiderocol resistance mechanisms, particularly those not directly linked to β-lactamase activity or traditional iron-uptake regulation.

The heatmap-based sequence homology and structural alignment revealed that several vitamin B12- and iron-associated TBDRs in *A. baumannii*, including BtuB_3, PirA, and CirA, share significant homology with the well-characterized *E. coli* BtuB and *K. pneumoniae* CirA receptors. This striking variability among TBDRs suggests that vitamin B12 and iron transport receptors may have co-evolved to meet distinct environmental and nutritional demands. Notably, predicted 3D structural overlays showed that *A. baumannii* BtuB_3 and *E. coli* CirA display conserved β-barrel architectures. This structural conservation, particularly within the extracellular loops and plug domains, provides a plausible basis for competition at the ligand-binding level. Molecular docking simulations further substantiated this hypothesis, indicating that vitamin B12 and cefiderocol share overlapping binding pockets within CirA and PirA receptors, raising the possibility that vitamin B12 could inhibit cefiderocol uptake by competitively occupying shared binding regions. These findings raise important questions about functional redundancy and receptor plasticity within the TBDR family in *A. baumannii*, underscoring the need for further studies to expand structural profiling and TBDR binding assays to validate the present observations and assess whether vitamin B12 exposure may ultimately compromise cefiderocol efficacy in clinical settings.

The transcriptomic analysis conducted on two genetically distinct CRAB strains with different basal cefiderocol MIC values revealed a strain-specific response to vitamin B12, particularly in metabolic pathways, stress response, and cellular adaptation pathways, with a similar response in key genes involved in methionine metabolism and vitamin B12 and iron uptake. The differences in the metabolic cellular response between AB5075 and AMA17 showed that AB5075 induces a metabolic reprogramming characterized by enhanced sulfur metabolism, increased expression of nutrient transporters, activation of oxidative stress defenses, and upregulation of biofilm-associated structures. Genes associated with protein synthesis, energy metabolism, and lipid biosynthesis and metabolism are strongly downregulated, suggesting a shift toward a lower metabolic state leading to the emergence of SCVs. SCVs, which produce colonies 5–10 times smaller in diameter than the parental strain, have been identified across numerous bacterial genera (57–59), with *Staphylococcus aureus* being the most extensively studied. The emergence of SCVs is considered a common adaptive response within the bacterial life cycle to counteract adverse environmental conditions such as physical stress, nutrient limitation, or antibiotic exposure (57, 59, 60). SCVs represent a slow-growing subpopulation characterized by distinct pathogenic and phenotypic traits (57, 61). Most SCVs share hallmark features, including reduced colony size, atypical morphology, delayed growth, and altered biochemical properties, typically involving downregulation of virulence gene expression alongside upregulation of cell wall-associated genes (57–59, 62). Their reduced susceptibility to antibiotics compared to wild-type strains enables SCVs to persist and contribute to chronic, relapsing infections (58, 62). Despite increasing research, the molecular mechanisms driving SCV formation and maintenance remain incompletely understood (57, 59, 61, 62).

Notably, opaque and translucent colony variants linked to differences in virulence, motility, and biofilm formation have been described and well characterized in *A. baumannii* AB5075 (63–65); however, there have not been reports describing the cellular processes involved in the formation of SCVs. Overall, the observed changes in the transcriptional response, confirmed by associated phenotypes, indicate that vitamin B12 can act as a regulatory signal modulating stress resistance, nutrient acquisition, and biofilm formation, enhancing the bacterium’s survival in hostile environments. In contrast to AB5075, AMA17 showed marked vitamin B12-mediated transcriptional changes in genes coding for translation and protein biosynthesis, together with stress tolerance, a cellular adaptation response. Another metabolic difference between the two strains was the differential expression of the *hut* operon (histidine catabolism); while AB5075 repressed it, AMA17 upregulates the expression of this operon. This may indicate strain-specific utilization of nitrogen sources under stress or altered carbon flow under B12 influence.

The transcriptomic analysis revealed the consistent downregulation of TonB-dependent transporters, including B12 and iron-siderophore receptors, in response to vitamin B12 exposure in both strains, supporting the idea that vitamin B12 could act as a signal, triggering negative feedback to limit further uptake of cofactors. However, this effect also includes iron receptors, a response that could affect the bacterial susceptibility to siderophore-antibiotics that use this entry route, such as cefiderocol. The possibility that vitamin B12 exposure leads to reduced TonB-dependent uptake, enhancing cefiderocol MIC values, was confirmed in a collection of clinical and model *A. baumannii* strains.

We cannot discard the possible contribution of alternative resistance mechanisms. For instance, AMA17 exhibited broad downregulation of outer membrane porins, with 15 out of 20 downregulated. Such a reduction in membrane permeability could restrict passive entry of antibiotics and represents a known resistance mechanism in gram-negative bacteria (13, 52, 66–70). Mutations in outer membrane proteins in other species have been linked with increased cefiderocol MICs (67, 68). In addition, the regulation of efflux pumps under vitamin B12 exposure can also be another factor contributing to cefiderocol resistance. This is not unexpected in isolates with intrinsically elevated baseline MICs, such as those harboring β-lactamases like NDM, which inherently reduce susceptibility to cefiderocol (13, 15, 19, 31, 41, 71, 72). In this context, the phenotypic increase in MICs upon co-exposure to vitamin B12 and cefiderocol appears to be primarily driven by NDM overexpression, compounded by porin loss. These changes likely act synergistically with only modest modulation of TBDR expression, collectively enhancing the hydrolytic capacity of the existing β-lactamase repertoire.

From a clinical perspective, this strain-to-strain heterogeneity indicates that vitamin B12 will not uniformly antagonize cefiderocol across all CRAB isolates. Rather, our data support the possibility that cefiderocol efficacy is most likely to be compromised in high-risk, highly resistant strains, such as *bla*_NDM_-harboring isolates with intrinsically elevated cefiderocol MICs, which are commonly encountered in critically ill patients.

In representative isolates, co-exposure to vitamin B12 and cefiderocol leads to increased MICs for cefiderocol, particularly pronounced in *A. baumannii*, where certain forms of vitamin B12, such as methylcobalamin, produced more marked changes. Across different assays, co-exposure to vitamin B12 was associated with elevated cefiderocol MICs compared to baseline, reduced bactericidal activity, and the emergence of subpopulations with SCV phenotypes, indicative of adaptive survival responses. However, at high concentrations of both cefiderocol and vitamin B12, the emergence of SCVs could suggest a possible convergent adaptive mechanism involving a shift toward a low-energy state, further contributing to the observed resistance phenotype. The phenotypic resistance acquired following co-exposure to cefiderocol and vitamin B12 appears to be stable and irreversible. This not only poses a potential risk to the treated patient by compromising therapeutic efficacy but also raises concern about the dissemination of cefiderocol resistance to other patients, including those without prior exposure to either compound.

The level of resistance to cefiderocol was proportional to the concentration of vitamin B12 used. This finding may have significant clinical implications, particularly in septic patients, where elevated baseline levels of vitamin B12 have been documented (73). Micronutrients such as vitamin B12 are increasingly being investigated as adjunctive therapies in refractory vasodilatory shock due to their pleiotropic effects. Although the exact mechanisms by which intravenous vitamin B12 mitigates catecholamine-resistant vasodilation remain unclear, cobalamins have been shown to scavenge nitric oxide and hydrogen sulfide, thereby modulating systemic inflammation and contributing to the anti-inflammatory response (74, 75). In line with these effects, a high-dose intravenous hydroxocobalamin (5 g) has been evaluated in phase 2 trials for patients with septic shock and post-operative vasoplegia, where it was associated with a reduction in vasopressor requirements compared to placebo (74, 76). However, such pharmacological doses would result in serum concentrations that far exceed the levels shown in our study to induce irreversible increases in cefiderocol MICs. This raises concern that therapeutic use of high-dose vitamin B12, particularly in critically ill patients, may inadvertently contribute to cefiderocol resistance development and treatment failure (77).

Importantly, our work does not propose withholding vitamin B12 from patients who require replacement. Rather, our data show that, in cefiderocol-treated patients who also receive a parenteral clinical dose of vitamin B12 supplementation, the timing, dose, and route of vitamin B12 administration should be considered and that future pharmacodynamic models and susceptibility testing protocols may need to incorporate physiologically relevant vitamin B12/iron conditions.

Our *in vitro* experiments showing antagonism, MIC elevation, and selection of small-colony variants were consistently observed at methylcobalamin concentrations above approximately 25–40 mg/L in checkerboard, time-kill, and population analysis profile assays. Thus, while the highest methylcobalamin dose in our experiments (400 mg/L) does not attempt to mimic typical plasma levels, the threshold concentrations at which we observed clinically concerning phenotypes lie well within the spectrum of transient supraphysiological exposures which can plausibly be reached in critically ill patients receiving a parenteral clinical dose of vitamin B12.

Beyond individual drug-nutrient co-administration, our findings may have implications at the hospital and population levels. *Acinetobacter* is well known for its ability to persist in healthcare environments, particularly in low- and middle-income countries with limited infection prevention and control. Even if vitamin B12-driven selection of cefiderocol-resistant derivatives is rare, a single successful clone emerging in a cefiderocol-treated patient receiving clinically indicated parenteral vitamin could subsequently disseminate to other patients. Consistent with this concern, vitamin B12-selected mutants in our study maintained elevated cefiderocol MICs over serial passages without exogenous vitamin B12, indicating that the resistance phenotype is stable once acquired. While we focused on modeling acute high-dose parenteral supplementation typical of critically ill patients, chronic exposure to lower vitamin B12 concentrations is common in other settings, as patients with diabetes treated long-term with metformin, those on chronic dialysis, individuals after bariatric surgery, and strict vegans, among others. Whether such chronic, moderate exposures can similarly shape *Acinetobacter* colonization dynamics and subtly enrich cefiderocol-less-susceptible subpopulations over time remains unknown.

Our present work may have some limitations. First, we acknowledge that the mechanisms underlying strain-specific responses to vitamin B12 remain incompletely understood, likely reflecting the complex genomic plasticity of *A. baumannii*. While two genetically distinct CRAB strains were analyzed in depth, the study of additional strain types from diverse backgrounds will be necessary to generalize our observations. Second, although our structural and docking data support substrate overlap between cefiderocol and vitamin B12 within TBDRs, experimental validation of direct binding through biochemical assays will be needed. Third, the role of other B12 isoforms and the interplay with additional environmental signals, such as iron levels, remains unexplored. Furthermore, *in vivo* validation using animal infection models is essential to confirm the relevance of vitamin B12-induced cefiderocol resistance in clinical settings. Future studies should also investigate the transcriptional regulators mediating this response and explore whether targeted modulation of vitamin B12 pathways can restore cefiderocol efficacy. Finally, clinical surveillance studies assessing vitamin B12 serum levels in critically ill patients receiving cefiderocol could clarify the potential clinical translational implications of our findings.

In conclusion, our study identifies a previously unrecognized mechanism by which a common micronutrient, vitamin B12, alters cefiderocol efficacy in *A. baumannii* by promoting TBDR-mediated competition and affecting iron homeostasis, thereby increasing MICs, altering time-kill responses, and favoring persistence/SCV phenotypes. These effects were dose-dependent, strain-specific, and amplified by host-like fluids and are consistent with structural/docking evidence for overlapping binding sites between B12 and cefiderocol on key receptors. The findings suggest that the host’s nutrient status is a critical variable in siderophore-antibiotic performance and may help explain certain *in vivo* treatment failures.

## MATERIALS AND METHODS

### Bacterial strains

The *E. coli* K12 reference strain harboring a wild-type plasmid encoding *bla*_NDM-5_ was previously exposed to HPF to evaluate cefiderocol susceptibility. Under HPF exposure, this strain developed subpopulations with elevated cefiderocol MICs (*E. coli* K12 NDM-5 IHC A and B). These resistant colonies were subsequently isolated and subjected to whole-genome sequencing to investigate potential adaptive or resistance-associated changes. For transcriptomic analyses, two well-characterized CRAB model strains, AB5075 and AMA17 (Table S2) (78–80), were selected based on their distinct clonal lineages, resistance genotypes, and clinical origins across different regions and years. Ten total CR *Acinetobacter* spp. strains, including CDC reference strains and clinical isolates (79–81) (Table 3), were used for cefiderocol susceptibility assays under B12 exposure and additional treatments. The susceptible model strains ATCC 17978, ATCC 19606, and A118 (82, 83), along with the quality control strains *E. coli* ATCC 25922 and *P. aeruginosa* ATCC 27853 strains, were also used. In addition, a panel of carbapenem-resistant gram-negative pathogens, including five *K. pneumoniae*, three *E. coli*, and three *P. aeruginosa* isolates, two of which were CDC AR Bank reference strains (CDC AR Bank #0356 and CDC AR Bank #0064), was included to further assess the effect of vitamin B12 on cefiderocol susceptibility (Table S7).

### Whole-genome sequencing

Genomic DNA from *E. coli* K12 NDM-5 and the isogenic derivatives IHC A and IHC B was extracted using the Wizard Genomic DNA Purification Kit (Promega, Madison, WI, USA). The same procedure was used to extract genomic DNA from *A. baumannii* strain AR Bank #0033 and its derivative, obtained after 24 h exposure to vitamin B12 and cefiderocol. Whole-genome sequencing was performed using the NovaSeq X Plus platform (Illumina), generating 2 × 151 bp paired-end reads. Raw sequencing quality was assessed with FASTQC (https://www.bioinformatics.babraham.ac.uk/projects/fastqc/), and adapter trimming and quality filtering were carried out using Trimmomatic (v.0.40; parameters: ILLUMINACLIP: TruSeq3-PE.fa:2:30:10, LEADING:3, TRAILING:3, SLIDINGWINDOW:4:15, MINLEN:36) (84). SPAdes (v.3.15.4, default parameters) (85) was used for *de novo* genome assembly, the quality of which was evaluated using QUAST (v.5.2.0) (86). Genome annotation was performed using PROKKA (v.1.14.5) (87), and SNV analysis was done with breseq (v.0.38.1) in consensus mode (88). Gene content analysis between AB5075 and AMA17 was performed using Roary software. Comparative genomic analyses of the IHCA and IHCB derivatives versus the *E. coli* K-12 parental strain, as well as of the *A. baumannii* AR Bank #0033 wild-type strain versus its derivative, obtained after 24 h exposure to vitamin B12 and cefiderocol, were used to detect mutations and other genomic changes potentially associated with the increased cefiderocol MIC phenotype. All sequencing data, including FASTQ files, assembled genomes, and annotations, have been deposited in Zenodo (https://zenodo.org/records/17859083, accessed 8 December 2025).

### TonB-dependent receptor comparative analyses

FASTA format files from *A. baumannii*, *E. coli*, and *K. pneumoniae* were used to perform sequence alignments and phylogenetic analysis of protein sequences of TBDRs involved in vitamin B12 and iron uptake. These included annotated receptors such as BtuB, CirA, PiuA, and PirA. Multiple sequence alignment was performed using the MAFFT software (89), and phylogenetic trees were constructed using the maximum likelihood method using IQTree2. Pairwise sequence similarities were further quantified by calculating BLAST *E* values (90, 91), which were overlayed onto the clustering results and visualized in a heatmap format. To study the structural homology of selected TBDRs, predicted 3D structures of *A. baumannii* BtuB_3 (UniProt ID: A0A0D5YDN7) and *E. coli* CirA (UniProt ID: P17315) were retrieved from the AlphaFold Protein Structure Database (92). Visual molecular dynamics (93) was used to verify that protein receptors did not include water molecules. Structural alignments were performed using “realign with TM-align” (94) within the NCBI iCn3D viewer (95, 96) to compare the A chains of each receptor. Structural overlays were visualized by two approaches: color by structure (to distinguish receptor identity) and color by pLDDT (to assess model confidence). RMSD and TM-align (94) scores were recorded to evaluate structural similarity. To examine the binding of vitamin B12 to BtuB_3 and PirA, docking complexes for vitamin B12 with *A. baumannii* BtuB_3 and PirA were exported from HotSpot Wizard (97, 98) and visualized using the RCSB Mol* 3D Viewer (99). Residue contacts within 3 Å of the ligand were determined using UCSF ChimeraX (100). Each ligand was selected under the “models” tab, followed by the “select zone” command to identify interacting side chains within the cutoff radius. This allowed for the identification of residues contributing to B12 recognition across different TonB-dependent receptors. Finally, the ligand-protein docking and binding site analyses of cefiderocol and vitamin B12 to TBDRs were performed using the HotSpot Wizard (v.3.1) platform (97, 98), which integrates AutoDock Vina (101). AlphaFold-predicted receptor models were screened using Fpocket (102) within HotSpot Wizard (97, 98) (probe radius 2.8 Å, minimum probe radius 1.4 Å, clustering threshold 3.5 Å) to identify binding pockets. Vitamin B12 ligand structures were obtained from the RCSB PDB (https://www.rcsb.org/ligand/B12), and cefiderocol was obtained from DrugBank (103). Ligands were converted to PDBQT format using Open Babel’s -opdbqt function (104). For each receptor, the docking grid encompassed the entire protein surface (exhaustiveness: 70, modes: 5, energy range: 10, and seed: 0). Docking energies were recorded and visualized with JSmol within HotSpot Wizard. Docking outputs depicting ligand poses and receptor structure files were combined in ChimeraX (100). Hydrogen bonds, hydrophobic interactions, B-factors, electrostatics, and contacts were applied to the joined files, then loaded into LigPlot+ (105) for further binding site analysis.

### RNA extraction and quantitative RT-qPCR

*A. baumannii* AB5075 and AMA17 were cultured in LB broth with agitation for 18 h at 37°C. Overnight cultures were then diluted 1:10 in fresh LB broth or LB broth supplemented with vitamin B12 (methylcobalamin, 100 mg/L) and incubated with agitation for 18 h at 37°C. The Direct-zol RNA Kit (Zymo Research, Irvine, CA, USA) was used for RNA extraction from three independent culture samples. RNA samples were DNase-treated (Thermo Fisher Scientific, Waltham, MA, USA) following the manufacturer’s instructions. Samples were confirmed to have no DNA contamination through PCR amplification of the 16S rDNA gene. RNA sequencing was outsourced to SeqCenter (SeqCenter LLC, Pittsburgh, PA, USA). Ribosomal RNA depletion was done using the Ribo-Zero kit (Illumina), and the construction of the cDNA library was performed with the TruSeq Stranded Total RNA Library Prep kit (Illumina) from three independent replicates per sample. Analysis of the quality of the Illumina reads, trimming of low-quality bases, and removal of Illumina adapters were performed as described previously (106).

Reads were aligned to the cognate of *A. baumannii* AB5075 and AMA17 genomes using the Burrows–Wheeler Alignment software (v.0.7.17) and visualized using the Integrative Genomics Viewer. Read counts per gene were calculated using FeatureCounts (107), and differential gene expression analysis was performed using DESeq2. DEGs were defined as those displaying an FDR-adjusted *P* value of <0.05 and a log_2_ fold change of >1. The RNA-seq data generated in the current study are available in the NCBI repository with the GEO accession GSE307121.

qRT-PCR was performed to confirm RNA-seq data and analyze differential gene expression. cDNA was prepared using the iScript Reverse Transcription Supermix for qRT-PCR (Bio-Rad, Hercules, CA, USA). Quantitative PCR was performed using qPCRBIO SyGreen Blue Mix Lo-ROX (PCR Biosystems Ltd, London, UK) per manufacturer’s recommendations, respectively. Primers used to confirm RNA-seq results and the expression of genes associated with virulence and antimicrobial resistance are listed in Table S10. Experiments were performed in technical and biological triplicates. The results were analyzed using the qBASE method (108) using *secA* and *rpoB* genes as normalizers (31). Data are presented as normalized relative quantities. Data differences were determined by two-way ANOVA followed by Tukey’s multiple-comparison test (*P* < 0.05) using GraphPad Prism (GraphPad software v.10.0.0, San Diego, CA, USA).

### Antimicrobial susceptibility testing

The MICs of cefiderocol were determined using broth microdilution (BMD) assays with ID-CAMHB following the Clinical and Laboratory Standards Institute (CLSI) guidelines. Two forms of vitamin B12 were used in these assays, obtained from different vendors: methylcobalamin (Sigma-Aldrich, Combi-Blocks, and from nutritional supplements Doctor’s Best) and cyanocobalamin (Sigma-Aldrich or Supelco). Vitamin B12 was added to ID-CAMHB at defined concentrations (0–400 mg/L) to evaluate its impact on cefiderocol susceptibility. For selected experiments, ID-CAMHB was supplemented with 3.5% HSA or 4%–10% HPF to simulate host-like conditions. When applicable, commercial E-strips for cefiderocol (Liofilchem S.r.l., Roseto degli Abruzzi, Italy) were used in parallel for comparative purposes and identification of heteroresistance or resistant mutants. All procedures were performed according to CLSI (109) and EUCAST guidelines (https://www.eucast.org/clinical_breakpoints) and interpreted using the most current breakpoints available. *E. coli* ATCC 25922 and *P. aeruginosa* ATCC 27853 served as controls. Checkerboard assays were conducted in 96-well microtiter plates to assess the interaction between cefiderocol and methylcobalamin. Twofold serial dilutions of cefiderocol (0.25–256.0 mg/L) were combined with serial dilutions of methylcobalamin (0–400 mg/L) in ID-CAMHB. After inoculation with ~5 × 10⁵ CFU/mL of a bacterial suspension, plates were incubated at 37°C and inspected visually after 18–20 h incubation. “Trailing” in the wells test (multiple wells of tiny or faint growth relative to the growth control) was ignored. The FICI was calculated with the following formula: FICI = (MICAB / MICA), where A represents cefiderocol and B represents methylcobalamin. The methylcobalamin MIC was not included in the calculation, as it lacks intrinsic antibacterial activity. A FICI value of ≤0.5 indicates synergy; >0.5 and <4.0 indicate additivity; and >4 indicates antagonism. Time-kill kinetics were assessed to evaluate the bactericidal activity of cefiderocol in the presence and absence of methylcobalamin in three CRAB strains (AB5075, AR Bank #0033, and AR Bank #0056) and three additional gram-negative CR bacilli strains (Ec7499, Kp01, and PAE319). All bacterial isolates were grown to mid-log phase, diluted to ~5 × 10⁵ CFU/mL in ID-CAMHB, and exposed to cefiderocol at 1×, 2×, and 4× MIC, with or without 100 mg/L methylcobalamin. Bacterial suspensions were incubated at 37°C with shaking, and aliquots were collected at 0, 3, 6, and 24 h. Experiments were performed in three biological replicates. Samples were serially diluted, plated on Mueller–Hinton agar, and incubated for 18–24 h at 37°C for colony enumeration. Bactericidal activity was defined as a ≥3 log_10_ CFU/mL reduction in viable counts from the initial inoculum. Regrowth was monitored through the 24-h time point, and colonies from regrowth were subcultured in antibiotic-free media to assess the stability of resistance. Post-vitamin B12 exposure MICs were determined by BMD using ID-CAMHB as described above and interpreted using the most current breakpoints available. *E. coli* ATCC 25922 and *P. aeruginosa* ATCC 27853 served as controls. Checkerboard assays were conducted in 96-well microtiter plates to assess the interaction between cefiderocol and methylcobalamin. Twofold serial dilutions of cefiderocol (0.25–256.0 mg/L) were combined with serial dilutions of methylcobalamin (0–400 mg/L) in ID-CAMHB. After inoculation with ~5 × 10⁵ CFU/mL of a bacterial suspension, plates were incubated at 37°C and inspected visually after 18–20 h incubation. Trailing in the wells test (multiple wells of tiny or faint growth relative to the growth control) was ignored. The FICI was calculated with the following formula: FICI = (MICAB / MICA), where A represents cefiderocol and B represents methylcobalamin. The methylcobalamin MIC was not included in the calculation, as it lacks intrinsic antibacterial activity. A FICI value of ≤0.5 indicates synergy; >0.5 and <4 indicates additivity, and >4 indicates antagonism. Time-kill kinetics were assessed to evaluate the bactericidal activity of cefiderocol in the presence and absence of methylcobalamin. *A. baumannii* CDC AR Bank #0033 was grown to mid-log phase, diluted to ~5 × 10⁵ CFU/mL in ID-CAMHB, and exposed to cefiderocol at 1×, 2×, and 4× MIC, with or without 100 mg/L methylcobalamin. Bacterial suspensions were incubated at 37°C with shaking, and aliquots were collected at 0, 3, 6, and 24 h. Experiments were performed in three biological replicates. Samples were serially diluted, plated on Mueller–Hinton agar, and incubated for 18–24 h at 37°C for colony enumeration. Bactericidal activity was defined as a ≥3 log_10_ CFU/mL reduction in viable counts from the initial inoculum. Regrowth was monitored through the 24-h time point, and colonies from regrowth were subcultured in antibiotic-free media to assess the stability of resistance. Post-vitamin B12 exposure MICs were determined by BMD using ID-CAMHB as described above.

### Cellular and phenotypic analysis

The cell morphology and cellular structure of AB5075 and SCVs 1–3 were examined by scanning electron microscopy (SEM). A standardized inoculum prepared from overnight cultures was used. The cells were harvested, washed, and fixed in 2% glutaraldehyde. Samples were prepared on protamine-coated glass slides, dehydrated through an ethanol series, and critical point dried for SEM imaging with a JCM-7000 NeoScope.

Biofilm assays were performed using AB5075 and AMA17 as previously described (31, 52). Cells were cultured in LB broth and incubated in static conditions at 37°C for 24–48 h. All experiments were conducted in triplicate, with at least three technical replicates per biological replicate. Statistical analyses were performed using GraphPad Prism. *P* values of <0.05 were considered statistically significant.

The resazurin reduction assay was used to evaluate the metabolic activity of selected AB5075 small-colony variants obtained under vitamin B12 exposure (110). Standardized inocula (10⁻³ inoculum) of overnight cultures of AB5075 (used as control) and the small-colony variants SCV1, SCV2, and SCV3 were used. Suspensions were added to sterile 96-well plates, and then resazurin solution was added to test wells, excluding growth controls, to reach a final assay volume of 100 μL. Growth and negative controls included media with and without resazurin. Plates were incubated at 37°C for 2 h in the dark, and color changes were monitored: blue indicated no activity; purple indicated intermediate activity; and pink indicated high metabolic activity. Fluorescence (excitation 530–560 nm, emission 590 nm) and absorbance (570 and 600 nm) were measured using a microplate reader (Multimode Microplate Reader, BioTek Synergy H1, Agilent), with independent experimental triplicates performed under reduced light conditions due to resazurin’s light sensitivity.

## Data Availability

PDB files, structural/docking PDB files for TBDRs, and FASTQ files of *E. coli* NDM5 wild type and IHC derivatives, plus *A. baumannii* CDC AR Bank #0033 (M27835) and a derived mutant, have been deposited in the Zenodo repository (https://zenodo.org/records/17859083). The RNA-seq data generated in the current study are available in the NCBI repository with the GEO accession number GSE307121.

## SUPPLEMENTAL MATERIAL

Supplemental material mbio.03760-25-s0001.docx Supplemental figures and Table S10.

Table S1 mbio.03760-25-s0002.xlsx Whole-genome sequencing analysis.

Table S2 mbio.03760-25-s0003.docx Characteristics of the selected CRAB strains.

Table S3 mbio.03760-25-s0004.xlsx Predicted ligand binding sites for vitamin B12.

Table S4 mbio.03760-25-s0005.xlsx Comparative genomic analysis of strains AB5075 and AMA17.

Table S5 mbio.03760-25-s0006.xlsx Comprehensive RNA-seq data set listing all DEGs.

Table S6 mbio.03760-25-s0007.xlsx List of the 52 DEGs shared between strains AB5075 and AMA17 after vitamin B12 exposure.

Table S7 mbio.03760-25-s0008.docx MICs of cefiderocol.

Table S8 mbio.03760-25-s0009.docx Cefiderocol MICs in the presence of increasing concentrations of methylcobalamin.

Table S9 mbio.03760-25-s0010.docx Cefiderocol MICs for selected CRAB strains.

ASM does not own the copyrights to Supplemental Material that may be linked to, or accessed through, an article. The authors have granted ASM a non-exclusive, world-wide license to publish the Supplemental Material files. Please contact the corresponding author directly for reuse.
