# Supplementary material for: Vitamin B12 promotes cefiderocol resistance and small-colony variants in carbapenem-resistant Acinetobacter baumannii
Source: mBio. 2026 Jan 16;17(2):e03760-25. doi: 10.1128/mbio.03760-25 (PMC12892962; doi:10.1128/mbio.03760-25)
Supplement: Supplemental material — Supplemental figures and Table S10. [file mbio.03760-25-s0001.docx]

**Supplementary Figures and Table S10**


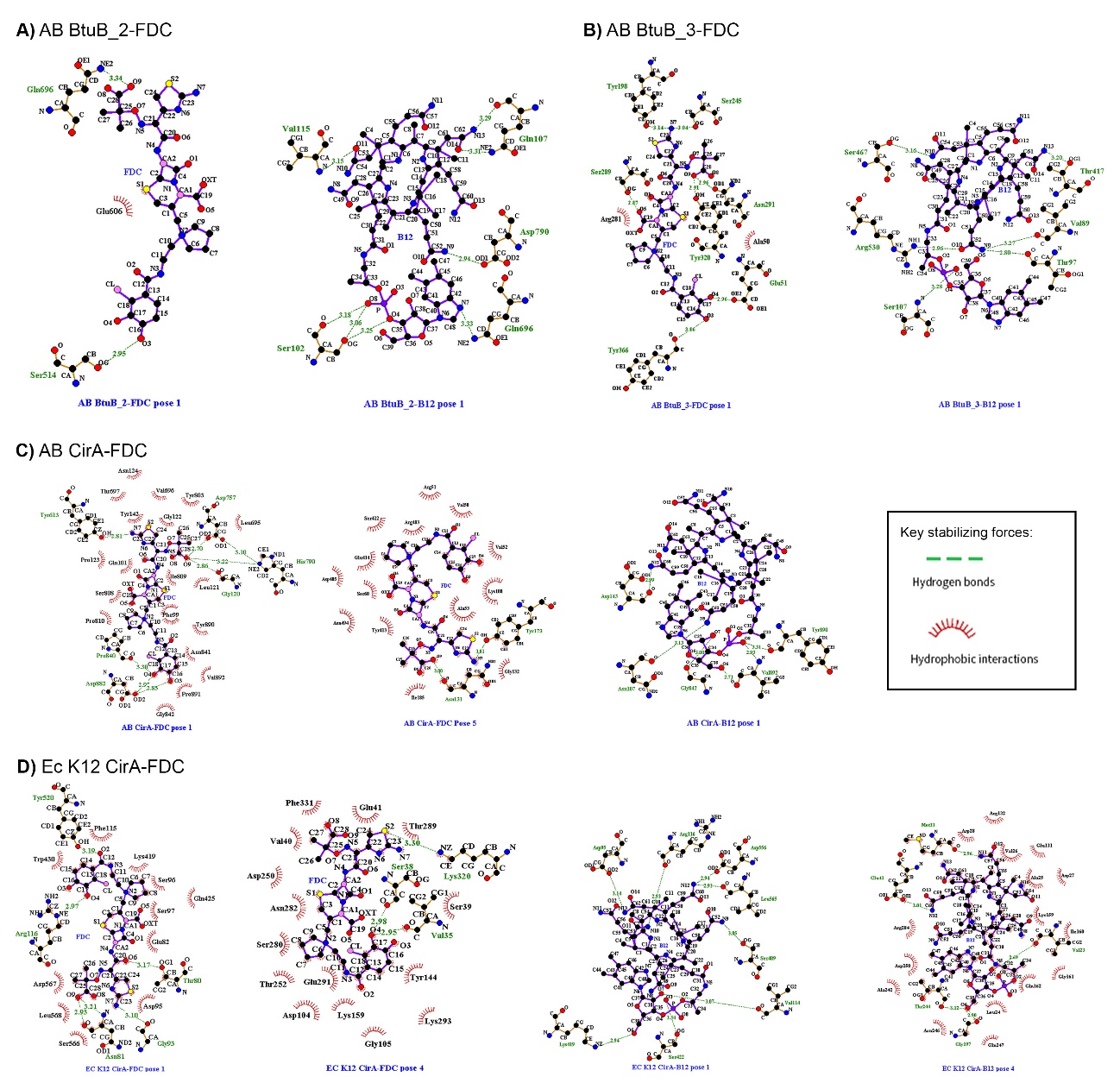


**Figure S1. Predicted binding interactions between cefiderocol (FDC) or vitamin B12 and TonB-dependent receptors from *A. baumannii* and *E. coli*.** Two-dimensional LigPlot+ representations of the predicted interactions between either FDC or vitamin B12 (B12) and TonB-dependent receptors (TBDRs) from *A. baumannii* (BtuB_2, BtuB_3, CirA) and *E. coli* K12 (CirA). Ligand-protein docking was performed using AutoDock Vina via the HotSpot Wizard v3.1 platform. Receptor structures were obtained from the AlphaFold Protein Structure Database, and ligand structures from DrugBank (FDC) and the RCSB PDB (B12). Binding pockets were identified with Fpocket, and docking simulations were run using an exhaustiveness of 70 across the entire receptor surface. The best-ranked binding poses (by affinity) were selected for visualization. Receptor-ligand interactions within 3 Å were identified in UCSF ChimeraX and visualized in LigPlot+, showing hydrogen bonds (green dashed lines) and hydrophobic interactions (red arcs). Each panel illustrates the primary binding mode for FDC and B12, highlighting key stabilizing residues across different TBDRs.


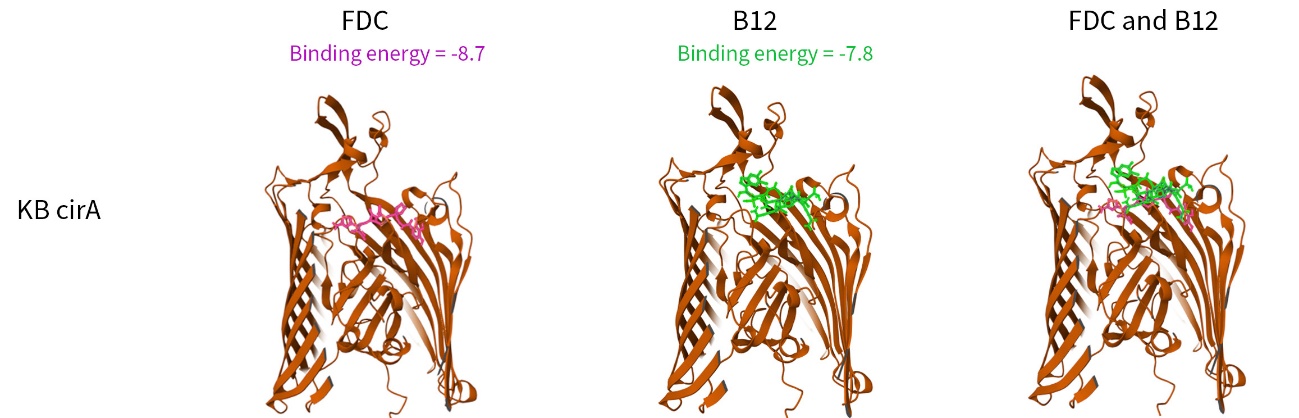


**Figure S2. Molecular docking of *Klebsiella pneumoniae* CirA with cefiderocol (FDC) and vitamin B12 (B12).** The predicted structure of CirA (orange, AlphaFold, UniProt P17315) was used for docking with FDC and B12 using HotSpot Wizard v3.1 with AutoDock Vina. The corresponding predicted binding energies (kcal/mol) for FDC and B12 are indicated in pink and in green, respectively. Left: CirA–FDC complex. Centre: CirA–B12 complex. Right: Combined docking shows partially overlapping binding sites, suggesting competitive binding. Ligand poses were visualized with ChimeraX.


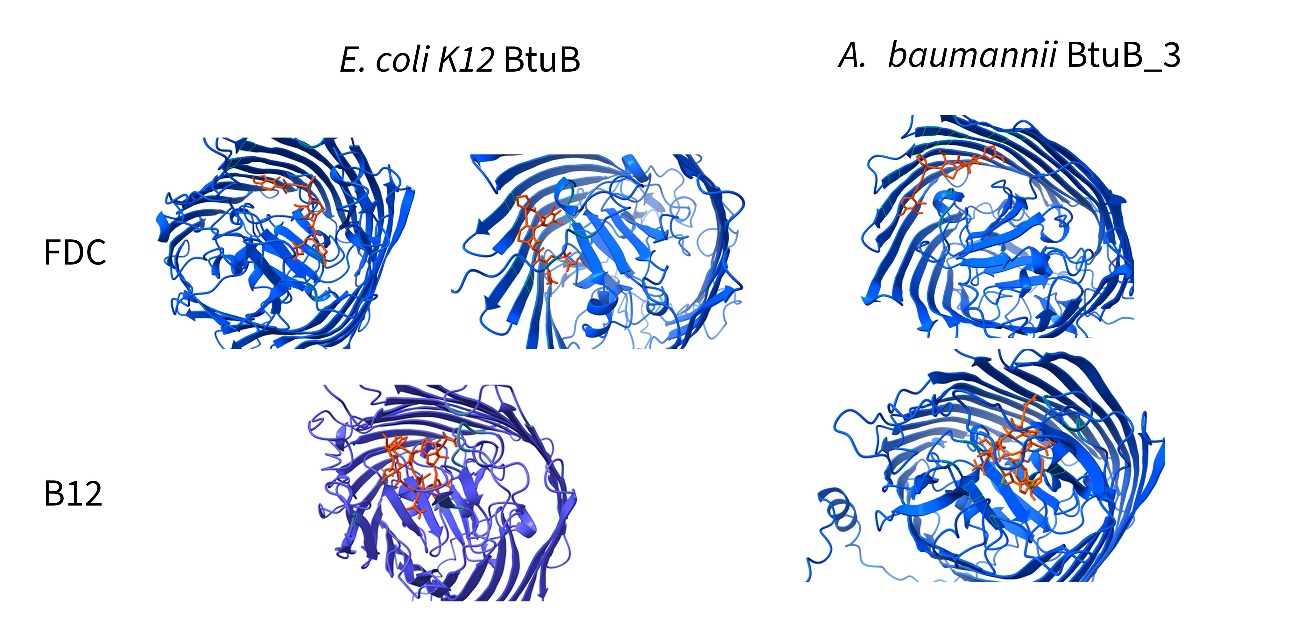


**Figure S3. Predicted docking poses of cefiderocol (FDC) and vitamin B12 (B12) in TonB-dependent receptors BtuB from *E. coli* and *A. baumannii*.** Docking complexes of FDC (top row) and B12 (bottom row) bound to *E. coli* K12 BtuB (left) and *A. baumannii* BtuB_3 (right), shown as cartoon representations. Receptor structures were obtained from the AlphaFold Protein Structure Database and prepared by removing water molecules using VMD. Ligands were retrieved from DrugBank (FDC) and the RCSB PDB (B12) and converted to PDBQT format with Open Babel. Docking was performed using AutoDock Vina through the HotSpot Wizard v3.1 platform, with a global search grid (exhaustiveness: 70). Ligand poses were visualized using UCSF ChimeraX. Orange sticks represent ligand atoms; protein chains are colored by secondary structure. Representative binding conformations were selected based on the lowest predicted binding energy and proximity of residues within 3 Å. These results highlight differences in binding pocket architecture and ligand accommodation between the BtuB orthologs from the two species.


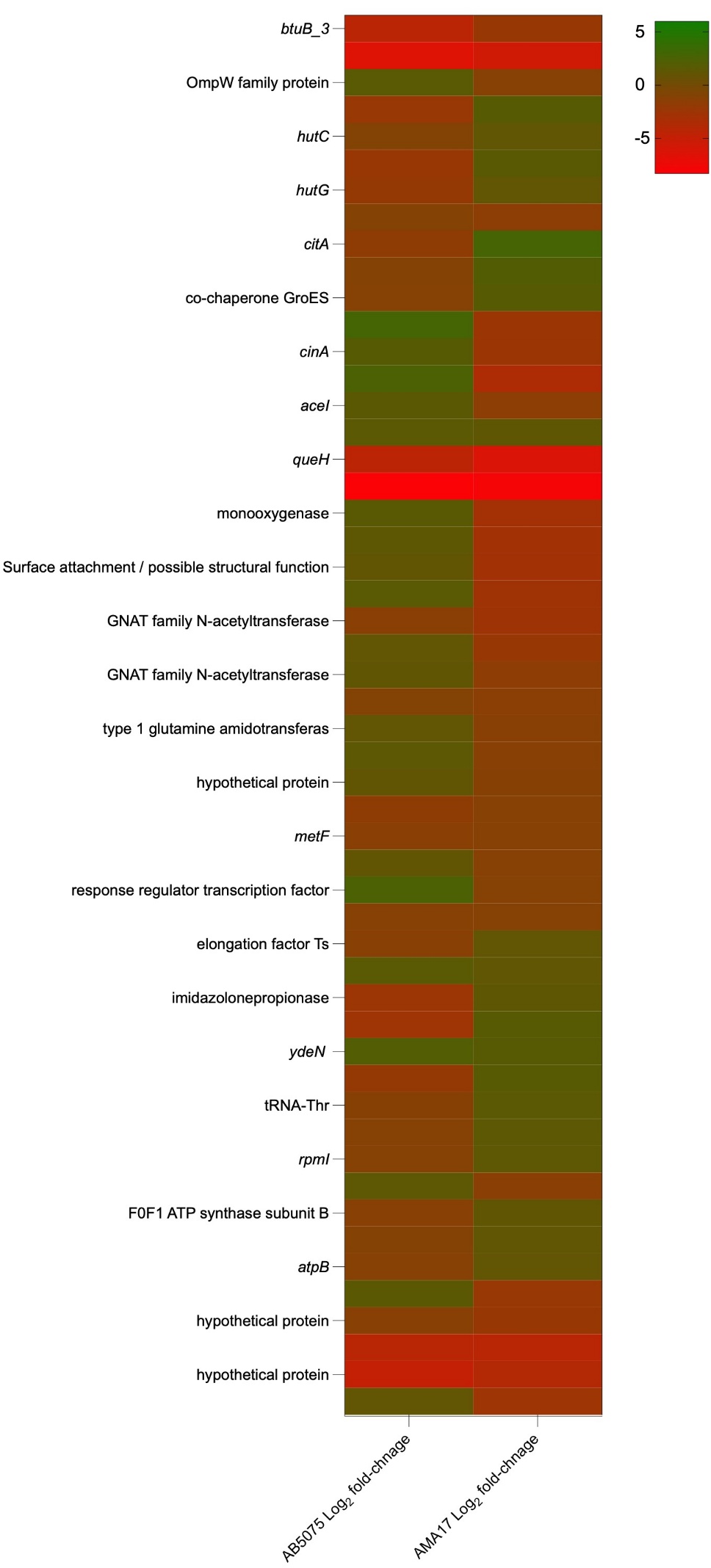


**Figure S4.** Heatmap showing the 52 differentially expressed genes (DEGs) shared between *A. baumannii* strains AB5075 and AMA17 after vitamin B12 supplementation.


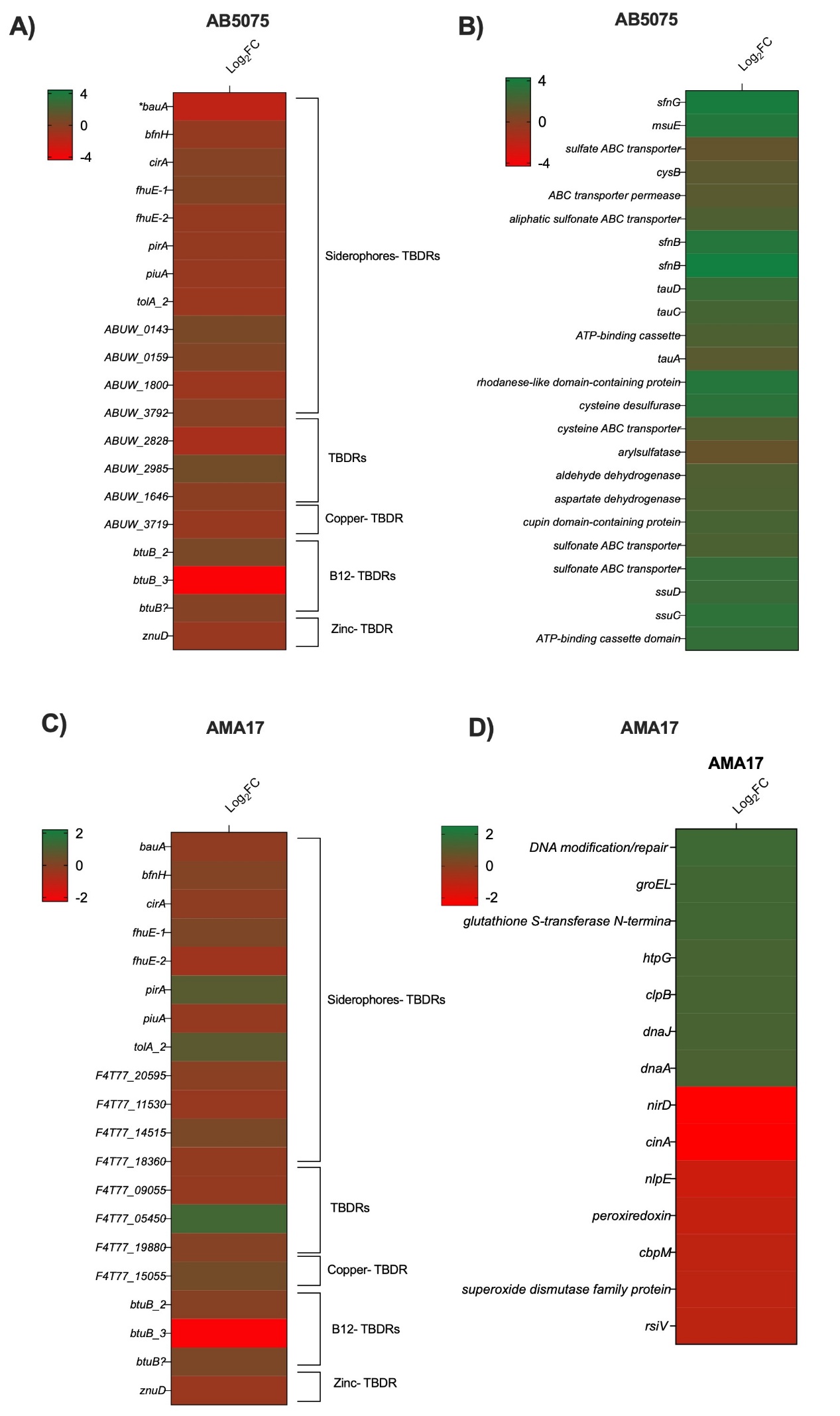


**Figure S5.** A) Heatmap showing downregulation of the acinetobactin siderophore gene cluster and other TonB-dependent receptors (TBDRs) in *A. baumannii* AB5075 following vitamin B12 exposure. B) Upregulation of sulfur metabolism-related genes in AB5075, including *sfnG, sfnB, msuE*, and taurine/sulfonate transporters (*tauD, ssuD, ssuC*). C) Expression profile of TBDRs in AMA17 after B12 exposure. D) Differential expression of stress response genes in AMA17, including upregulation of heat shock proteins (*groEL, dnaA, dnaJ, htpG, clpB*) and oxidative stress-related genes, alongside downregulation of selected stress tolerance genes (*nirD, cinA*).


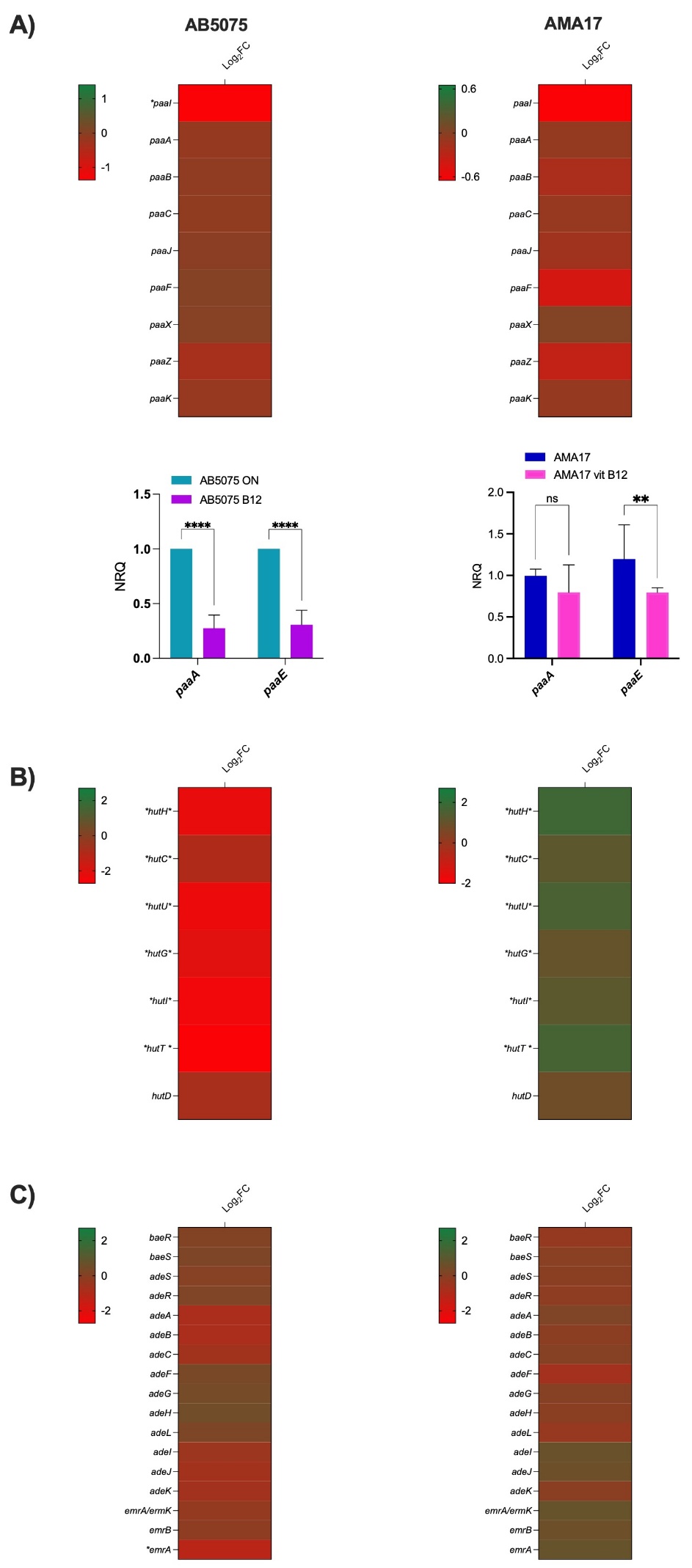


**Figure S6.** A) Downregulation of phenylacetic acid (*paa*) catabolic pathway genes in both *A. baumannii* strains AB5075 and AMA17 following vitamin B12 exposure determined by RNA-seq (top) and by qRT-PCR (bottom). The data presented are the mean ± standard deviation (SD) of normalized relative quantities (NRQ) derived from transcript levels calculated using the qBASE method. Statistical significance (P < 0.05) was determined by one-way ANOVA followed by Tukey’s multiple-comparison test. **, P < 0.01; ***, P < 0.001; ****, P < 0.0001; ns, not significant. Technical triplicates using three independent biological samples were used to collect experimental data. Error bars represent the standard deviation (SD). B) Divergent transcriptional regulation of the *hut* operon (histidine catabolism): strong downregulation in AB5075 (log₂ fold changes -0.8 to -2.7) and upregulation in AMA17 (log₂ fold changes +0.79 to +1.76). C) Differential expression of efflux pump genes and associated regulators in AB5075 and AMA17, including components of the AdeABC, AdeFGH, AdeIJK systems, and Emr family transporters.


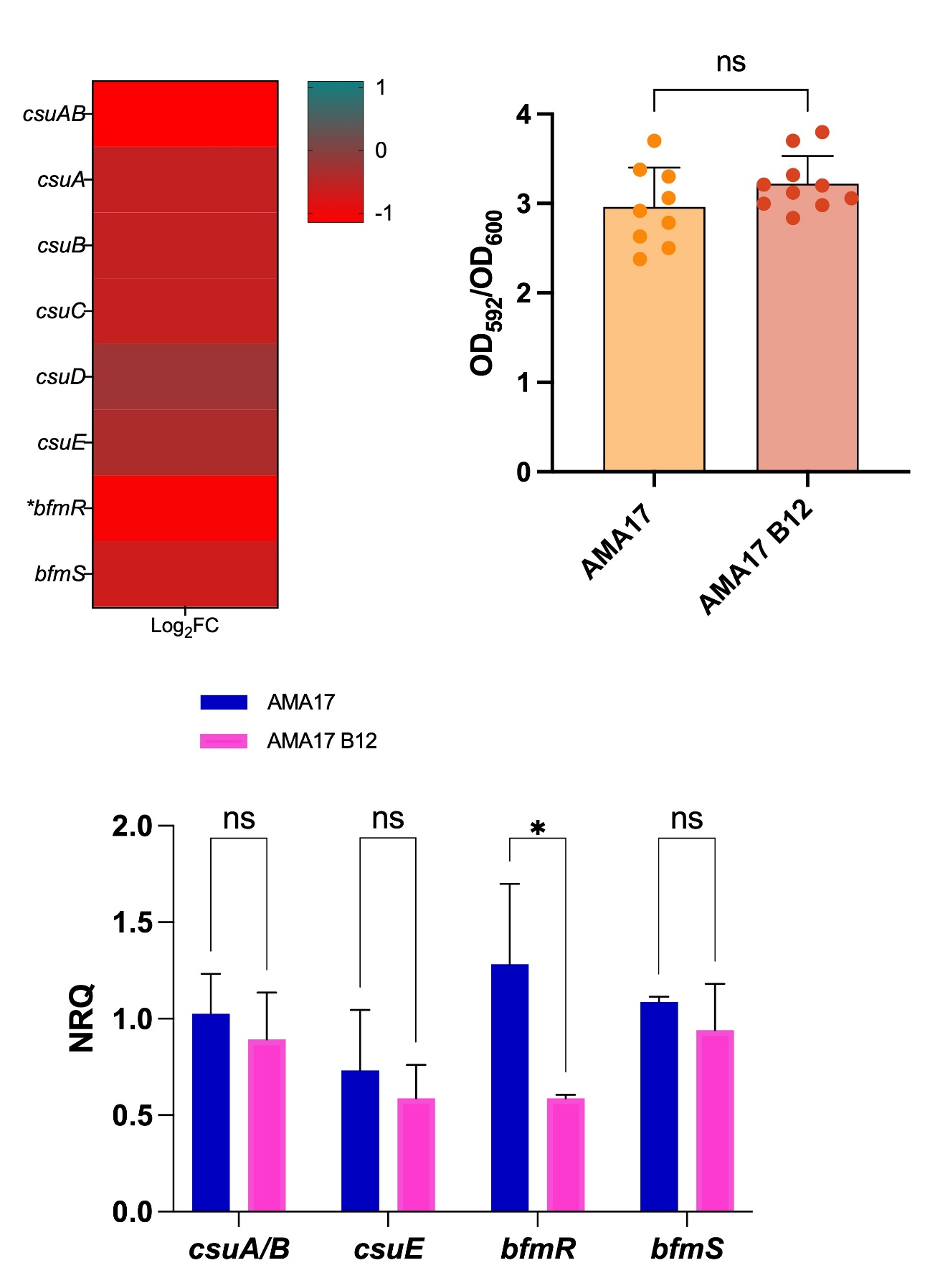


**Figure S7.** RNA-seq data and qRT-PCR analysis of the *csu* pili gene cluster showing no significant change in expression upon B12 exposure, consistent with unchanged biofilm formation measured by crystal violet assay.


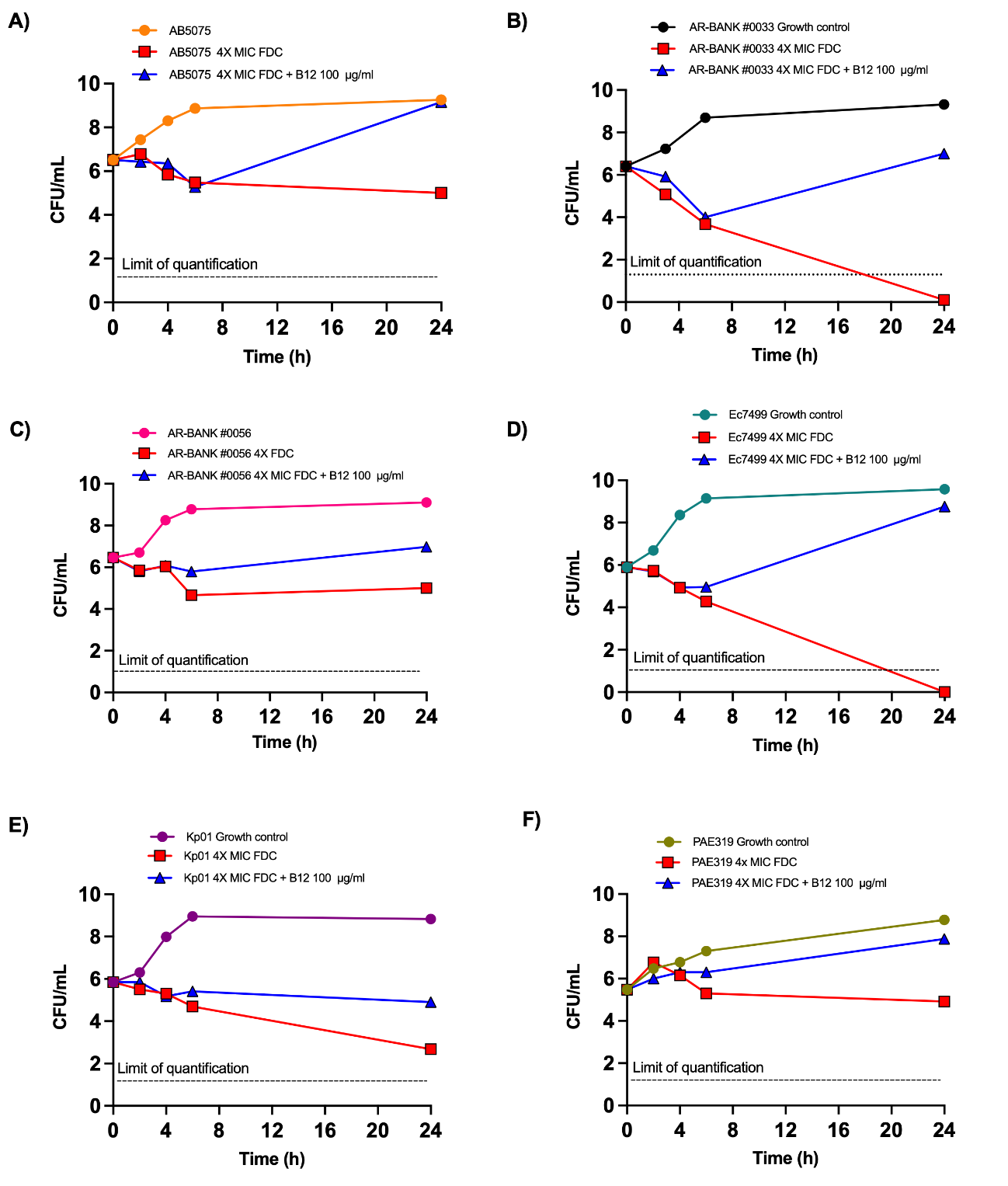


**Figure S8.** **Time-kill curves for six carbapenem-resistant Gram-negative strains exposed to cefiderocol at 4× MIC in the absence and presence of methylcobalamin (100 mg/L).** Bactericidal activity was evaluated over 24 h for *A. baumannii* AB5075 (A), *A. baumannii* CDC AR-BANK #0033 (B), *A. baumannii* CDC AR-BANK #0056 (C), *E. coli* Ec7499 (D), *K. pneumoniae* Kp01 (E), and *P. aeruginosa* PAE319 (F).

**Table S10 - Primers used in this work**

| **Primers** | **Sequence (5’–3’)** |
| --- | --- |
| secAqPCRF | ACTTGTTCGTGAACATGCTGGGTG |
| secAqPCRR | TGCTTCTGCTTGTTGCTGCTGT |
| rpoBF.rt | CAGAAGTCACGCGAAGTTGAAGGT |
| rpoBR.rt | AACAGCACGCTCAACACGAACT |
| NDM1-5F | ACAAGATGGGCGGTATGGAC |
| NDM1-5R | AATGAGCTGCACAGTGGGAA |
| qPCR_AB_amvA_Fw | ACGATTGATGCAACGGTGATG |
| qPCR_AB_amvA_Rv | GGCAAAATCAAACCCGCCAT |
| qPCR_AB_aceI_Fw | TCCAAGAGAAGACTCATTCATGCA |
| qPCR_AB_aceI_Rv | TGTTCCGGTCACTTCCATCG |
| atpA_qPCR_Fw | ATCCATCCGAGATCAGTGCG |
| atpA_qPCR_Rv | GCATCAGCAAGACCGTGAAT |
| qPCR_AB_atpB_Fw | ACATGCCCTCACTTCGACAG |
| qPCR_AB_atpB_Rv | AAGGCCAATTGACCAACCCA |
| atpD_qPCR_Fw | GGTGTTGTTCGTACCATCGC |
| atpD_qPCR_Rv | CGTCCATGATACGGCCAAGA |
| qPCR_AB_atpF_Fw | TGAAGTTTGTTTGGCCACCAC |
| qPCR_AB_atpF_Rv | CATCGGCAAGGTCAGCTTTC |
| nuoH_qPCR_Fw | TGTTGTTCTTGTTGCTGCGT |
| nuoH_qPCR_Rv | GGAACATACCACCAGGACCA |
| nuoL_qPCR_Fw | ACCTGTTCGTTGCAAGCATG |
| nuoL_qPCR_Rv | AAATGCTTTGATCGCTGCCC |
| nuoM_qPCR_Fw | TGTTGGCACATGGTTTGTCA |
| nuoM_qPCR_Rv | CCACGAAGACCACCCATCAA |
| csuAB F | CAGGCTGTACTGTAGGTG |
| csuAB R | CAGGATCTGTTCCGTCAC |
| *csuE* F | TGGACAAAGTGTATCGCCGG |
| *csuE* R | ACACCCCGATTCCCACAATC |
| bfmR F | CGATGGTAACCGTGCAATTCGT |
| bfmR R | ATCGTCTGCACCCATTTCCAGA |
| bfmSqPCRF | AAATCCGACAGGTGCGTTATGC |
| bfmSqPCRR | ATACGTGCCACAGGTGTTCTGA |
| bauA F | AAATGTTTGGCCGCGTTGAGGT |
| bauA R | CAATCGTGCAAACGGTTCATCAGC |
| basD F | TGCACAGATTGCTCCCGTGGTATT |
| basD R | ACTTGCGGCCCTTGTGAAATGA |
| basB F qPCR | CGGAAATGTATGAAAAGCT |
| basB R qPCR | TGTTGCTGTAACGCATTGGC |
| basA F | TACAGCTTTGGCATGTGCGCTT |
| basA R | TCACCACAACATCTGCCCGACTAT |
| barA F | TGCAACTTTTACTTTCAGGCGG |
| barA R | CATTGGTCTGGTCGAGTGCA |
| barB F | TTTGGATAGTCGGGGCGGCT |
| barB R | TGATGAGCAATTTGACGACGT |
| piuAF_qPCR | ATGGCGCAAGAAGCAGTTTC |
| piuAR_qPCR | TGTCTTTGAGAGGAGCCACG |
| pirAF_qPCR | GTCTATGGCTTTTGCTGCACA |
| pirAR_qPCR | GCGATTGCTTCACTTGCTCT |
| qPCR_btuB_AB_2_F | TGTTCGATCTCAGGCAACCC |
| qPCR_btuB_AB_2_R | AATGGGGGAAGCACTTTGTG |
| qPCR_AB btuB_3_F | CTACTCGTCTGGTAGGGGCT |
| qPCR_AB_btuB_3_R | AGCCGGAACTTCACTTGCTT |
| paaE F | CGGGTGAAATCCGCCGTTGTTAT |
| paaE R | ACTTCAAGCACATCGCCCACTT |
| paaA F | CAAATGCCTGATGCCTACCG |
| paaA R | TGGTGCACGAGTAATCCAGT |
| qPCR_AB_yiaD_Fw: | TGGTAGGGGCAGCAGTAGTA |
| qPCR_AB_yiaD_Rv: | CTGCGCCGATCAAAGTACCT |
| qPCR_AB_soxR_Fw: | TAACGGTTGGTGAGGTTGCA |
| qPCR_AB_soxR_Rv: | AACGGCGTTGGTTACCATTG |
| qPCR_AB_pcaJ2_Fw | GAAACTCAGCCGTGACCAGA |
| qPCR_AB_pcaJ2_R v | AGCTGGCAATCTTGGTTGGT |
| qPCR_AB_cpo_Fw | CACGCGATGCCCAAGTTTTA |
| qPCR_AB_cpo_Rv | GCGTCTGTCATGAGCGACTA |
| qPCR_AB_cinA_1_Fw | TTTTGAAAGTGCCTCTGCGG |
| qPCR_AB_cinA_1_Rv | TTCCTTTACGCGAAGGTCGT |
| carO F | GGCGGATGAAGCTGTTGTTC |
| carO R | GCCATAACAAAGCACCACCG |
| ompAqPCRF | GCTGCTAATGCTGGCGTAAC |
| ompAqPCRR | GCCGTCTACGTCGCCTTTAA |
