# Supplementary material for: Vitamin B12 promotes cefiderocol resistance and small-colony variants in carbapenem-resistant Acinetobacter baumannii
Source: mBio. 2026 Jan 16;17(2):e03760-25. doi: 10.1128/mbio.03760-25 (PMC12892962; doi:10.1128/mbio.03760-25)
Supplement: Table S2 — Characteristics of the selected CRAB strains. [file mbio.03760-25-s0003.docx]

**Table S2.** Characteristics of the selected CRAB strains AB5075 and AMA17.

| CR-Strain | Origin | Clonal lineage | Year of isolation | Geographical location | *bla* genes* |
| --- | --- | --- | --- | --- | --- |
| AB5075 | Tibia/osteomyelitis | International Clone I (IC1) | 2008 | USA | *bla*_OXA23_ |
| AMA17 | Blood | ST25 (emergent clone) | 2016 | Argentina | *bla*_NDM-1_ |

* carbapenemases
