# Supplementary material for: Vitamin B12 promotes cefiderocol resistance and small-colony variants in carbapenem-resistant Acinetobacter baumannii
Source: mBio. 2026 Jan 16;17(2):e03760-25. doi: 10.1128/mbio.03760-25 (PMC12892962; doi:10.1128/mbio.03760-25)
Supplement: Table S7 — MICs of cefiderocol. [file mbio.03760-25-s0008.docx]

**Table S7.** Minimum inhibitory concentrations (MICs) of cefiderocol (FDC) in the presence or absence of vitamin B12 (methylcobalamin^#^) supplementation in other Gram-negative bacilli.

|  |  |  | |  | | |  | | FDC MIC (mg/L) |
| --- | --- | --- | --- | --- | --- | --- | --- | --- | --- |
| Strains | | | Carbapenemases | | ID-CAMHB | | | ID-CAMHB + B12^#^ (100 mg/L) | |
| Kp27278 (*K. pneumoniae)* | | | *bla*_NDM-5_/*bla*_KPC-2_ | | 1 | | | 8 | |
| Kp01 (*K. pneumoniae)* | | | *bla*_NDM-5_ | | 0.5-1 | | | 4 | |
| KpJAF1(*K. pneumoniae)* | | | *bla*_NDM-1_ | | 0.5 | | | 4 | |
| KPZBE2 (*K. pneumoniae*) | | | *bla*_NDM-5_ | | 0.5 | | | 4 | |
| KPZCA8 (*K. pneumoniae*) | | | *bla*_NDM-5_ | | <=0.25 | | | 4 | |
| Ec7499 (*E. coli)* | | | *bla*_KPC-2_ | | 0.125 | | | 1 | |
| EcZBB4 (*E. coli)* | | | *bla*_KPC-2_ | | | 0.06 | | 0.25 | |
| EcZAU4 (*E. coli)* | | | *bla*_NDM-1_ | | | 2 | | 8 | |
| PAE319 (*P. aeruginosa*) | | | *bla*_KPC-5_ | | | 0.125 | | 1 | |
| PAE27829÷ (*P. aeruginosa*) | | | *bla*_SPM-1_ | | | <=0.25 | | 4 | |
| PAE27875∞ (*P. aeruginosa*) | | | *bla*_KPC-2_ | | | 2 | | 8 | |

^#^Sigma-Aldrich (C_63_H_91_CoN_13_O_14_P), ÷ CDC AR-Bank #0356, ∞ CDC AR-Bank #0064
