# Supplementary material for: Vitamin B12 promotes cefiderocol resistance and small-colony variants in carbapenem-resistant Acinetobacter baumannii
Source: mBio. 2026 Jan 16;17(2):e03760-25. doi: 10.1128/mbio.03760-25 (PMC12892962; doi:10.1128/mbio.03760-25)
Supplement: Table S8 — Cefiderocol MICs in the presence of increasing concentrations of methylcobalamin. [file mbio.03760-25-s0009.docx]

**Table S8**. Cefiderocol MICs in the presence of increasing concentrations of methylcobalamin in CS *A. baumannii* ATCC strains

| Cefiderocol MIC mg/L (BMD) | | | |
| --- | --- | --- | --- |
| Strain | ID-CAMHB | ID-CAMHB +Methylcobalamin^#^ (100 mg//L) | ID-CAMHB +Methylcobalamin^#^ (200 mg/L) |
| ATCC 17978 | 0.005 | 0.2 | 0.4 |
| ATCC 19606 | 0.125 | 0.25 | 1 |

^#^ Sigma-Aldrich (C_63_H_91_CoN_13_O_14_P)
