# Supplementary material for: Vitamin B12 promotes cefiderocol resistance and small-colony variants in carbapenem-resistant Acinetobacter baumannii
Source: mBio. 2026 Jan 16;17(2):e03760-25. doi: 10.1128/mbio.03760-25 (PMC12892962; doi:10.1128/mbio.03760-25)
Supplement: Table S9 — Cefiderocol MICs for selected CRAB strains. [file mbio.03760-25-s0010.docx]

**Table S9.** Cefiderocol MIC of selected CRAB strains exposed to B12 (methylcobalamin), 4% HPF, 10% HPF, and/or 3.5% HSA.

| **Cefiderocol MIC mg/L (BMD)** | | | | | | | | |
| --- | --- | --- | --- | --- | --- | --- | --- | --- |
| Strain | ID-CAMHB | ID-CAMHB + B12^#^ (100 µg/mL) | ID-CAMHB + HPF (4%) | ID-CAMHB + HPF (4%) + B12 (100 µg/mL) | ID-CAMHB + HPF (10%) | ID-CAMHB + HPF (10%) + B12 (100 µg/mL) | ID-CAMHB + HSA (3.5%) | ID-CAMHB + HSA (3.5%) + B12 (100 µg/mL) |
| AB5075 | 0.5-1 | 8 | 2 | 4 | 2 | 128 | 2 | 4 |
| AMA17 | 32 | >512 | 128 | >512 | 128 | >512 | 64 | >512 |
| AMA32 | 1 | 4 | >512 | >512 | >512 | >512 | 256 | >512 |

^#^ Methylcobalamin (Sigma-Aldrich), HPF: human pleural fluid (Innovative Research), HSA: huma serum albumin (Sigma-Aldrich).
